# A Plumieridine-Rich Fraction From *Allamanda polyantha* Inhibits Chitinolytic Activity and Exhibits Antifungal Properties Against *Cryptococcus neoformans*

**DOI:** 10.3389/fmicb.2020.02058

**Published:** 2020-08-28

**Authors:** Eden Silva e Souza, Vanessa de Abreu Barcellos, Nicolau Sbaraini, Júlia Catarina Vieira Reuwsaat, Rafael de Oliveira Schneider, Adriana Corrêa da Silva, Ane Wichine Acosta Garcia, Gilsane Lino von Poser, Euzébio Guimarães Barbosa, João Paulo Matos Santos Lima, Marilene Henning Vainstein

**Affiliations:** ^1^Bioinformatics Multidisciplinary Environment, Universidade Federal do Rio Grande do Norte, Natal, Brazil; ^2^Centro de Biotecnologia, Universidade Federal do Rio Grande do Sul, Porto Alegre, Brazil; ^3^Centro de Ciências Biológicas, Universidade Federal de Santa Catarina, Florianópolis, Brazil; ^4^Department of Pharmacy, Universidade Federal do Rio Grande do Sul, Porto Alegre, Brazil; ^5^Department of Pharmacy, Universidade Federal do Rio Grande do Norte, Natal, Brazil

**Keywords:** *Cryptococcus neoformans*, target prediction, chitinase, plumieridine, antifungal activity, drug discovery, glycoside hydrolase family 18

## Abstract

Cryptococcosis is a fungal infection caused mainly by the pathogenic yeasts *Cryptococcus neoformans* and *Cryptococcus gattii*. The infection initiates with the inhalation of propagules that are then deposited in the lungs. If not properly treated, cryptococci cells can disseminate and reach the central nervous system. The current recommended treatment for cryptococcosis employs a three-stage regimen, with the administration of amphotericin B, flucytosine and fluconazole. Although effective, these drugs are often unavailable worldwide, can lead to resistance development, and may display toxic effects on the patients. Thus, new drugs for cryptococcosis treatment are needed. Recently, an iridoid named plumieridine was found in *Allamanda polyantha* seed extract; it exhibited antifungal activity against *C. neoformans* with a MIC of 250 μg/mL. To address the mode of action of plumieridine, several *in silico* and *in vitro* experiments were performed. Through a ligand-based a virtual screening approach, chitinases were identified as potential targets. Confirmatory *in vitro* assays showed that *C. neoformans* cell-free supernatant incubated with plumieridine displayed reduced chitinase activity, while chitinolytic activity was not inhibited in the insoluble cell fraction. Additionally, confocal microscopy revealed changes in the distribution of chitooligomers in the cryptococcal cell wall, from a polarized to a diffuse cell pattern state. Remarkably, further assays have shown that plumieridine can also inhibit the chitinolytic activity from the supernatant and cell-free extracts of bacteria, insect and mouse-derived macrophage cells (J774.A1). Together, our results suggest that plumieridine can be a broad-spectrum chitinase inhibitor.

## Introduction

Cryptococcosis is a neglected fungal infection caused predominantly by *Cryptococcus neoformans* and *Cryptococcus gattii* (Kwon-Chung et al., 2017). *C. neoformans* is considered an opportunistic pathogen that infects mainly immunocompromised patients, while *C. gattii* is also capable of infecting immunocompetent individuals (Kwon-Chung et al., 2014). The infection occurs when dry cryptococci cells or spores are inhaled and reach the lungs, where they can either be controlled by the host immune system in case of an immunocompetent patient, or they may remain latent for a certain period (Giles et al., 2009; Velagapudi et al., 2009; Sabiiti and May, 2012; Ballou and Johnston, 2017). Under conditions of immunocompromise, the pathogen can disseminate from the lungs to the brain through different mechanisms and frequently cause fatal meningitis (Srikanta et al., 2014; Denham and Brown, 2018).

Cases of cryptococcosis are closely related to the pandemic events of AIDS, with less than 300 reports in the late 1950s and more than a million in 2006 (Park et al., 2009). The Joint United Nations Programme on HIV/AIDS (UNAIDS) estimates a mortality rate of 1.4 million AIDS patients annually, of which 15% are due to cryptococcosis alone (Rajasingham et al., 2017). Moreover, reports of cryptococcosis in immunocompetent patients are increasing (Chen et al., 2008; Suchitha et al., 2012; Poley et al., 2019).

*Cryptococcus* cells have two relevant therapy targets: the extracellular polysaccharide capsule and the cell wall. The cryptococci capsule is composed of the polysaccharides glucuronoxylomannan (GXM) and glucuronoxylomannogalactan (GXMGal), with trace amounts of mannoproteins (Bose et al., 2003; Wang et al., 2018). It has been observed that, specific antibodies against GXM interfere with capsular fibrils organization, potentially contributing to host defense (Martinez et al., 2004; Agustinho et al., 2018). However, no drug capable of impairing capsule production and formation has been developed so far. The cryptococcal cell wall is composed of β-linked glucans [β-(1,3) and β-(1,6)] and chitin, a linear polymer of β-1,4-*N*-acetyl-glucosamine (GlcNAc) that accounts for the rigidity and integrity of the cell wall (Banks et al., 2005; Gilbert et al., 2010; Agustinho and Nosanchuk, 2017). Echinocandins are important antifungal agents capable of interrupting the β-(1,3) glucan synthesis, being an effective treatment against several fungal infections (Denning, 2003). However, none of the discovered echinocandins are effective against *C. neoformans* (Feldmesser et al., 2000; Maligie and Selitrennikoff, 2005).

For hyphal branching and growth, autolysis, and morphogenesis, fungi employ several enzymes, such as glucanases and chitinases (Adams, 2004; Duo-Chuan, 2006; Ene et al., 2015). Chitinases are responsible for the hydrolysis of the chitin β-1,4 linkage resulting in monomers and oligomers of GlcNAc (Howard et al., 2003). These enzymes have been reported in a variety of organisms beside fungi, including plants, bacteria and humans (Grover, 2012; Junges et al., 2014; Rathore and Gupta, 2015). In *C. neoformans*, chitinases have been reported to be essential for sexual development but not vegetative growth and asexual reproduction (Baker et al., 2009). Furthermore, it has been suggested that cryptococcal chitinases are indirectly enrolled in modifications of the melanin scaffold (i.e., through the regular cell-wall remodeling activity) that lead to the detachment of melanin granules detachment from the cell wall (Camacho et al., 2019).

Chitinases can be classified into endochitinases and exo-chitinases. The endochitinases break chitin randomly at internal sites and release low molecular mass GlcNAc multimers (Sahai and Manocha, 1993). The exo-chitinases are divided into chitobiosidases (β-*N*-acetylhexosaminidase), which catalyzes di-acetylchitobiose starting at the non-reducing end of chitin, and 1-4-β-glucosaminidases, that are responsible for cleavage of endochitinase oligomeric products, generating GlcNAc monomers (Liu and Kokare, 2017).

Regarding classification, the Carbohydrate-active enzymes database (CAZy) classify chitin degrading enzymes into the Glycoside Hydrolase families (GH) 18, 19, and 20 (Lombard et al., 2014). GH18 and 19 enzymes are known as chitinases due to their ability to degrade chitin polymers, while GH20 enzymes cleave dimeric units of GlcNAc. The GH20 family comprises chitobiases and β-*N*-acetylhexosaminidases (Funkhouser and Aronson, 2007; Oyeleye and Normi, 2018). All fungi are reported to have chitinases of the glycoside hydrolase 18 family (GH18) (Oyeleye and Normi, 2018), excepting the parasitic fungus *Nosema bombycis*, which harbors GH19 chitinases (Han et al., 2016). On the other hand, chitinases from GH19 family are predominantly observed in plants (Udaya Prakash et al., 2010).

Several chitinase inhibitors have been described in the literature. For instance, the natural peptides argifin and argadin can inhibit *Aspergillus fumigatus*, *Serratia marcescens*, and human GH18 chitinases (Rao et al., 2005b). Caffeine was shown to be a chitinase inhibitor for the fungus *Clonostachys rosea* chitinase CrChi1. Also, conservation in the binding site is crucial for the effectiveness of this inhibitor (Yang et al., 2010). Methylxanthines, which includes caffeine, harbor anti-inflammatory properties and are also reported as GH18 chitinase inhibitors (Rao et al., 2005a). A chemical class named acetazolamide has been reported as a chitinase inhibitor in the pathogenic fungus *A. fumigatus* (Schüttelkopf et al., 2010). Noteworthy, several chitinolytic enzymes are not intracellular, which makes it possible to explore inhibitors that do not need to cross the cell wall and plasmatic membrane (Hamid et al., 2013).

The current treatment of cryptococcal meningoencephalitis consists of an induction, consolidation, and maintenance regimen (Mourad and Perfect, 2018a). The Infectious Disease Society of America (IDSA) recommends a 2-week treatment with amphotericin B (AMB) and flucytosine (5-FC) followed by treatment with fluconazole with time frame and dose depending on the patient’s response (Perfect et al., 2010). The combination of AMB and 5-FC shows more fungicidal activity than the sole treatment with AMB (Sloan and Parris, 2014). Although not always effective, this is still the best treatment available nowadays, which unfortunately, is not commercialized worldwide (Pappas, 2010). Additionally, the currently used drugs for cryptococcosis treatment have some disadvantages as *C. neoformans* strains are innately heteroresistant to fluconazole *in vitro*, even producing highly resistant subpopulation (Sionov et al., 2013). Thus, the administration of fluconazole as monotherapy (in case of AMB and 5-FC lack of availability) or at the end of the combination treatment may lead to occurrence of heteroresistant strains *in vivo* (Sionov et al., 2013). Furthermore, hepato- and nephrotoxicity are also reported during the cryptococcosis treatment, a side effect mostly caused by AMB administration (Krysan, 2015). Thus, there is a need for drugs with less or no toxicity but still effective against *Cryptococcus* spp. In this context, natural products are an interesting starting point for alternative treatment drugs and studies of natural compounds with anti-cryptococcal activity are increasing (da Silva et al., 2016; Teixeira et al., 2018).

The *Allamanda* genus (Apocynaceae: Gentianales) comprises 15 plant species distributed in South America (Sakane and Shepherd, 1986; The Plant List, 2020). Included in this genus, *Allamanda polyantha* is endemic of the Brazilian Atlantic Forest (Flora do Brasil, 2019). Plants of this genus are used in popular medicine to treat several illnesses, with potential antifungal, diuretic, antidiabetes and antiparasitic properties (Petricevich and Abarca-Vargas, 2019). Several iridoid compounds have been isolated from *Allamanda* spp., especially from *A. cathartica.* The iridoids found in *A. cathartica* include, but are not limited to, plumiericin, isoplumiericin, plumieride, and plumieride coumarate (Petricevich and Abarca-Vargas, 2019). However, the aglycone configuration of plumieride, plumieridine, has not been reported for the *Allamanda* spp. On the other hand, isolation of plumieridine was reported from *Plumeria obtusa*, another Apocynaceae (Saleem et al., 2011).

Recently, our research group identified anticryptococcal activity in the seed’s extract of *A. polyantha* Müll. Arg. This antifungal activity was attributed to a fraction rich in plumieridine (Bresciani et al., 2020). However, the target and mode of action of this potential antifungal agent are still unknown. To address the potential targets of plumieridine, a ligand based virtual screening was performed and indicated that it targets *C. neoformans* chitinases. Thus, several *in vitro* and *in silico* assays were employed to evaluate the mechanism of action of this molecule in *C. neoformans* chitinases. Furthermore, the activity of plumieridine against insect, bacteria, and mouse-derived macrophage chitinases was also evaluated.

## Materials and Methods

### Plumieridine Isolation and Purification

Plumieridine isolation was performed as recently published (Bresciani et al., 2020). Briefly, *A. polyantha* seeds were crushed in a kitchen blender. Crushed seeds were placed in contact with ultrapure water (10 g/20 mL) for 4 h, under agitation. The liquid suspension was centrifuged (for 10 min at 7168 × *g*). The resulting supernatant was filtered in filter paper and polypropylene prefilter (AP 25, Millipore). The aqueous extract was completely lyophilized at −50°C and 0.040 mbar (Christ Alpha 1-4 LD plus, Germany) and stored at –80°C. The lyophilized crude extract was subjected to silica gel column chromatography (70-320 mesh, Merck), using a gradient elution of dichloromethane: methanol (95:5 to 80:20) as the mobile phase to obtain plumieridine. The fractions were chromatographed over preparative TLC (20 cm × 20 cm, 0.5 mm layer, SiO_2_ F254 plates – Merck) using a mixture of dichloromethane:methanol (80:20) as eluent. Fractions were subjected to another chromatographic column as mentioned above to obtain the compound in a higher degree of purity. Fractions with antifungal activity were submitted to nuclear magnetic resonance (NMR) recorded in CD_3_OD, on a Varian spectrometer, operating at 400 MHz. Peaks of residual water were used as an internal standard in ^1^H NMR spectra and the solvent peak was used as an internal standard in ^13^C spectra (Gottlieb et al., 1997). Results were analyzed with MestraNova software (v. 6.0.2).

### Antifungal Susceptibility Assay

Minimum inhibitory concentration (MIC) of the plumieridine-rich fraction was determined against *C. neoformans* strain H99 according to the Clinical and Laboratory Standards Institute M27-A2 (NCCLS, 2002) and compared to amphotericin and fluconazole values. The compound was resuspended in Milli-Q water with 10% DMSO (stock concentration: 20 mg/mL; final concentration of DMSO in the experiments was usually less than 1%), and filtered before use (polyvinylidene difluoride filter, 0.22 μm pore size, Millipore). MIC assays were performed in 96-well plates (Corning®, Corning, NY, United States). Plumieridine was serially diluted, starting with 1 or 1.25 mg/mL, in RPMI 1640 (pH 7; Gibco® Life Technologies, Waltham, MA, United States) buffered with MOPS (Acros Organics, Geel, Belgium). Plates were incubated at 37°C for 72 h. To ensure reproducibility, MIC assays were performed with every new batch of the plumieridine-rich fraction and the obtained values were compared with previous results (Bresciani et al., 2020).

### Virtual Screening

To predict potential plumieridine targets, an *ad hoc* ligand based virtual screening approach was performed. The pharmACOphore software, which allows the alignment of active compounds, was used to search for similar ligands bound to proteins in the Protein Data Bank (PDB) (Hessler et al., 2010).

### Molecular Modeling and Docking

Chitinase sequences from *C. neoformans* previously identified by Baker et al. (2009) were retrieved from FungiDB (Basenko et al., 2018) under the access codes: CNAG_03412 (Chi2), CNAG_02598 (Chi21), CNAG_04245 (Chi22), and CNAG_02351 (Chi4). Evaluation of putative signal peptides, transmembrane helices, and conserved domains was performed with SignalP, TMHMM, and Conserved Domain Database (CDD), respectively (Möller et al., 2001; Almagro Armenteros et al., 2019; Lu et al., 2020). As the best-identity hit for *C. neoformans* chitinase sequences against potential PDB templates were around 30%, molecular models, for each chitinase, were created using different approaches. Sequences were modeled on SwissModel (Waterhouse et al., 2018), Phyre2 (Kelley et al., 2015), and Robetta server (Song et al., 2013). All models were evaluated on SwissModel Structure Assessment Tool and the best model for each chitinase was chosen based on Ramachandran-favored, Outliers, MolProbity Score, QMEAN, and Rotamer Outliers (Benkert et al., 2010). Chitinase models were later used for molecular docking and dynamics simulations.

Molecular docking of all four chitinases and plumieridine was simulated using AutoDock Vina (Trott and Olson, 2010), with the UCSF Chimera interface (Pettersen et al., 2004). The best ligand position was considered as the lowest energy pose, thus, plumieridine was manually docked in the same position in all *C. neoformans* chitinases. To infer the way plumieridine interacts with the active site of chitinases, two orientations were assayed arbitrarily named inward and outward.

### Molecular Dynamics Simulations

Molecular dynamics simulations were performed on complexes obtained from molecular docking using GROMACS 5 software (Van Der Spoel et al., 2005) with the aid of the CHARMM force field (Vanommeslaeghe et al., 2010). Plumieridine-chitinase complexes were placed inside a cubic box large enough to allow for a minimum of 1.0 nm of space from the protein to the box. The solvent properties were mimetic using the TIP3P water model. The system had the charge neutralized with the addition of ions at the physiological concentration (0.15 μM). Volume (NVT) and pressure (NPT) equilibrium simulations were geometrically optimized in the solvated system. During the simulation, the temperature was kept constant at 300°K coupling the system with a V-rescale thermostat with a 13-coupling time of 0.1 ps. The pressure was also kept constant at 1 bar with the Parinello–Rahman coupling algorithm. Molecular dynamics simulations were performed during 2600 ps. ensuring the stabilization of root-mean-square deviation (RMSD).

### Chitinase Activity and Inhibitory Assays

To evaluate the inhibitory activity of plumieridine on chitinases, a variety of models, such as *C. neoformans*, *Bacillus subtilis*, *Tenebrio molitor*, and mouse-derived macrophage cells (J774.A1) were employed. *C. neoformans* cells were grown on either YPD (1% yeast extract w/v, 2% dextrose w/v, and 2% Bacto peptone w/v) or YPGlcNAc (1% yeast extract w/v, 2% *N*-acetylglucosamine w/v, and 2% Bacto peptone w/v) on shaker for 24 h at 30°C. Both media were used to compare whether there is a difference in chitinase activity due to a change in the carbon source (Baker et al., 2009). After incubation, the culture was centrifuged (9000 × *g* for 10 min) and the supernatant was collected and lyophilized (secreted fraction). Lysis buffer (50 mM Tris-HCl pH 8.0, 20 mM EDTA, 200 mM NaCl, 1% SDS, and 1% Triton; 5 min of incubation at room temperature followed by vortex for 2.5 min) was used to release the proteins attached to the cell membrane. After centrifugation (9000 × *g* for 10 min), the supernatant was collected (soluble cell fraction) as well as the resulting pellet (insoluble cell fraction). All samples were lyophilized and solubilized in phosphate-buffered saline (PBS; 20 mg/mL). *B. subtilis* strain ATCC6633 was grown in Luria-Bertani (LB) broth (Sigma-Aldrich Co., St. Louis, MO, United States) medium at 37°C for 24 h. The supernatant was lyophilized and resuspended to the concentration of 20 mg/mL in PBS. This solution was used in the assay. For the insect model, eight and a half grams of whole *T. molitor* larvae were dried frozen and ground to a powder using liquid nitrogen. The powder was homogenized in PBS 1:2 (w/v) for 15 min under agitation at room temperature. *T. molitor* crude extract was centrifuged (9000 × *g* for 10 min) and filtered with qualitative filter paper (Unifil, Brazil). The resulting supernatant was lyophilized and resuspended in PBS (2 mg/mL). Lastly, J774.A1 cells, obtained from *Banco de Células do Rio de Janeiro* (BCRJ; accession number 0121) were cultured in DMEM (Dulbecco’s modified Eagle’s medium; Gibco® Life Technologies) supplemented with 10% heat-inactivated fetal bovine serum (FBS; Gibco® Life Technologies), 1 mM L-glutamine (Gibco® Life Technologies), 1 mM sodium pyruvate (Gibco Life Technologies), 1% non-essential amino acids (Gibco® Life Technologies) and incubated at 37°C and 5% CO_2_ for 3 days. After this step, the cell culture was centrifuged (9000 × *g* for 10 min) and the resulting supernatant was lyophilized and subsequently resuspended in PBS (20 mg/mL).

Chitinase activity assays were performed employing 4-methylumbelliferyl β-D-*N,N′,N″*-triacetylchitotrioside (Sigma-Aldrich Co.) as substrate (Boldo et al., 2009). A standard curve was created using 4-methylumbelliferyl (4MU) (Sigma-Aldrich Co.). The assays were performed in 96-well coated microplates (Greiner CELLSTAR® Sigma-Aldrich Co.) and consisted of 100 μL of McIlvaine buffer pH 6.0, 5 μL of the substrate (0.8 mM), and 10 μL of the sample. The reaction was incubated at 37°C for 30 min. The fluorescence was read at 355 nm excitation and 460 nm emission on SpectraMax I3. Inhibitory assays employed an increasing plumieridine concentration diluted in McIlvaine buffer pH 6,0. Plumieridine was added in the following concentrations: 0, 33, 100, 160, and 260 μg/mL in a final volume of 200 μL. Quantification of samples was performed according to relative fluorescent units (RFU), using the standard curve previously generated.

### Quantitative Real-Time PCR (qRT-PCR)

Fungi were grown in the same two media previously described for 4 h at 30°C and 200 rpm in the presence and absence of plumieridine using the sublethal dose determined through MIC (156 μg/mL). Total RNA extraction was performed using glass beads and Trizol treatment (Invitrogen, Carlsbad, CA, United States), following the manufacturer’s guidelines. cDNA was synthesized using 1 μg of DNase-treated RNA and ImProm-II™ Reverse Transcription System (Promega, Madison, WA, United States), following the manufacturer’s guidelines. Quantitative PCR reactions were conducted at a final volume of 20 μL, containing 2 μL of cDNA (4 ng/μL), 2 μL SYBR Green (1:1000) (Invitrogen), 0.1 μL dNTP (10 mM), 2 μL PCR buffer 10x, 1.2 μL MgCl_2_, 0.05 U Platinum Taq DNA Polymerase (Invitrogen), and 1 μL of each primer (5 pmol/μL). The experiments were carried out on an Applied Biosystems 7500 Fast Real-Time PCR System® with thermal cycling conditions set to an initial step at 95°C for 10 min, followed by 40 cycles at 95°C for 15 s, then 60°C for 15 s and, lastly, 72°C for 60 s. A melting curve analysis was performed at the end of the reaction to confirm the presence of a single PCR product. All experiments were performed using three independent cultures, and each cDNA sample was analyzed in triplicate for each set of primers. Data were normalized to levels of *ACT1*, which was included in each set of PCR experiments. Relative expression was determined using the 2^–Δ*Ct*^ method. The primers used can be found in Supplementary Table S1.

### Confocal Microscopy

Overnight cultures grown in YPD were washed with PBS, diluted to 10^6^ cells/mL in minimal media (15 mM glucose, 10 mM MgSO_4_.7H_2_O, 29.4 mM KH_2_PO_4_ 13 mM glycine and 3 μM thiamine), plated (1 ml/well) in triplicate in 24-well plates with the following concentration of plumieridine: 0, 156, 312, and 625 μg/mL, and incubated at 37°C and 5% CO_2_ for 72 h. Cells were washed with PBS and fixed with paraformaldehyde, followed by incubation with 5 μg/mL of Wheat Germ Agglutinin (WGA) conjugated with Alexa 488 (green) for 30 min at 37°C. Cells were washed again with PBS and incubated with 5 μg/mL Calcofluor white (blue) for 30 min at 37°C. As treatment with plumieridine reduces cell count, it was not possible to observe the impact of treatment with different drug concentrations in the same number of cells. Cell count was performed and a percentage ratio between the total cell count and cells with apparent phenotypes was calculated for all treatments.

### Time Kill Assay

Overnight cultures grown in YPD were washed with PBS and inoculated (10^5^ cells/mL) in RPMI 1640 buffered with MOPS with and without plumieridine supplementation (312, 625, and 1250 μg/mL). Cultures were incubated at 37°C and 200 rpm for 0, 2, 4, 8, and 24 h. After incubation, 100 μL of each sample was taken and diluted 1:10 with PBS. Thirty μL of the dilution was plated in YPD agar plates for colony forming unit (CFU) determination. The experiments were performed in biological and technical duplicates. Water and DMSO 1.25% were employed as control and control vehicle, respectively (Klepser et al., 2000).

### *In silico* Prediction of Rat Oral Toxicity

An *in silico* prediction of oral toxicity in rats (Lethal Dose 50 – LD_50_) was calculated by TEST (Toxicity Estimation Software Tool; version 4.2.1) based on plumieridine structure, using default parameters. TEST employs known experimental toxicity values and applies Quantitative Structure-Activity Relationship (QSAR), against internal and external datasets, to predict the toxicity values for the query structure (Martin et al., 2012).

### Statistical Analysis

All experiments were performed in biological triplicates. One-way analysis of variance (One-way ANOVA) was used to evaluate triplicates from the same experiment, while Two-way ANOVA was used to perform comparisons among experiments. All graphs were generated in Prism – GraphPad 8.0 (GraphPad Software, Inc., San Diego, CA, United States). Letters in the graphs indicate statistical significance between samples evaluated.

## Results

### Plumieridine Putatively Targets Chitinases

The virtual screening approach resulted in 14,993 predicted targets and, among these, 38 hits belonged to *A. fumigatus* (Table 1 and Supplementary Table S2). The results were ranked according to free energy and *A. fumigatus* chitinase B1 in complex with the tripeptide VR0 (PDB 3CHE) was considered the most likely plumieridine target (Full results for *A. fumigatus* predicted targets are available in Supplementary Table S2.). Although 38 hits were recovered from *A. fumigatus*, several ones found the same target, with only 19 unique entries. For instance, 4LNB and 4LNG were found two times, 3CHE, 1W9U, and 4C1Y were found three times, 1W9V and 3CHF were found four times, and 4D52 was found 6 times. Noteworthy, 14 of the 19 hits are from chitinase structures, pointing chitinases as promising plumieridine targets.

**TABLE 1:** Virtual screening results.

Protein	PDB ID	Ligand
Chitinase	3CHE	Tripeptide (VR0)
Chitinase	3CHD	Dipeptide (WRG)
Farnesyltransferase	4LNG	Farnesyldiphosphate and tipifarnib
Chitinase	2IUZ	C2-dicaffeine
Chitinase	2A3B	Caffeine

*Five of 38 hits from *A. fumigatus* (based on ligand molecular fingerprint). The most likely target, based on *A. fumigatus* bound ligands, is the complex chitinase B-Tripeptide (VR0) obtained from the pentapeptide argifin.*

### Plumieridine Interacts With Chitinase Catalytic Residues

Signal peptide prediction analysis indicates that the *C. neoformans* chitinases Chi4, Chi21, and Chi22 can be secreted, while Chi2 does not have a predicted signal peptide sequence (Supplementary Table S3). TMHMM analysis shows that only Chi2 has a transmembrane domain (Supplementary Table S3) that expands from residues 21–43, a feature previously observed by Baker et al. (2009).

To create the models for docking, the chitinases sequences from *C. neoformans* were searched against PDB templates. Since the best-identity hits for these sequences against available templates were around 30%, molecular models for *C. neoformans* chitinases were created using different approaches (SwissModel, Phyre2, and Robetta). The best models (all created by SwissModel) were selected based on parameters presented by the SwissModel Evaluation tool and all models presented 80% or more residues in Ramachandran favored areas. Notably, folding conservation in the active site of all four chitinases can be observed (Supplementary Figure S1).

**FIGURE 1:**
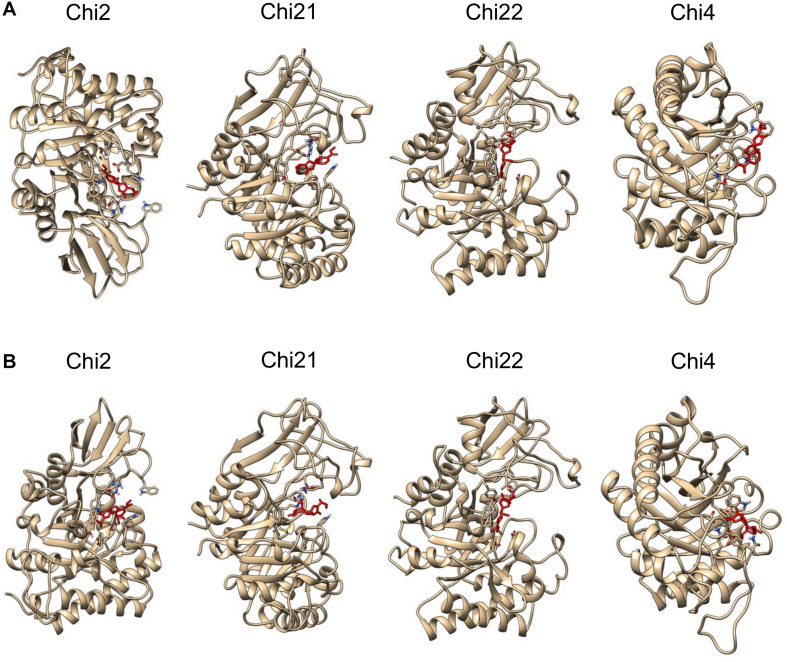
Plumieridine initial docked position. Plumieridine was manually docked employing the position of the bound ligand found through virtual screening analysis. The inward **(A)** and outward **(B)** orientations of plumieridine were evaluated for *C. neoformans* chitinases (Chi2, Chi21, Chi22, and Chi4). Plumieridine (red).

Molecular binding analysis indicates that plumieridine putatively interacts with amino acid residues in the chitinases active binding site (Figure 1). NCBI Conserved Domains analysis shows that *C. neoformans* Chi4 and Chi2 have a GH18_chitinase-like domain (cl10447), while Chi21 and Chi22 have a Glyco_hydro_18 domain (cl23725) (Supplementary Table S3). Both domains are part of the Glycoside Hydrolase 18 superfamily. This superfamily has the characteristic motif DxDxE observed through sequence alignment (Supplementary Figure S2) (Vaaje-Kolstad et al., 2004). In the binding simulations was observed that plumieridine interacts closely with the DxDxE motif. As these residues are directly involved in catalysis, the predicted interaction may be responsible for the loss in the chitinases catalytic activity.

### Plumieridine Inhibits Chitinase Activity in the Secreted and Cell-Soluble Fractions of *C. neoformans*

**FIGURE 2:**
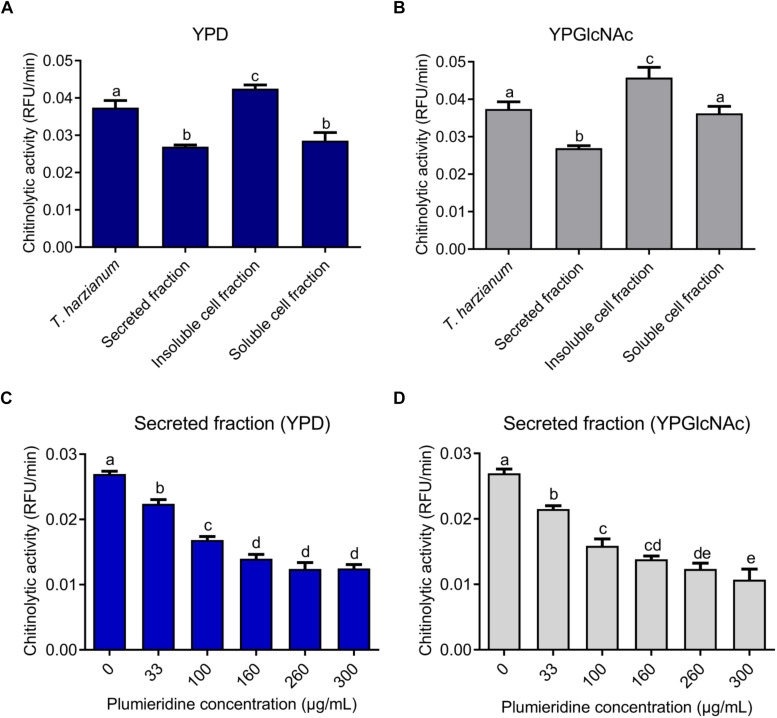
*Cryptococcus neoformans* chitinolytic activity. Chitinolytic activity of *T. harzianum* lysing enzymes and fractions of *C. neoformans* grown in YPD **(A)** and YPGlcNAc **(B).** Inhibition of chitinolytic activity by plumieridine treatment in the secreted fraction of *C. neoformans* grown in YPD **(C)** and YPGlcNAc **(D)**. Letters above bars indicate statistical significance among different concentrations.

Through NMR spectra, plumieridine was identified as the main compound present in the chromatographic fraction obtained from the seed extract of *A. polyantha* with anti-cryptococcal activity (Bresciani et al., 2020). To obtain enough plumieridine for all experiments, several batches of purification were needed. To ensure reproducibility, every new batch of the plumieridine-rich fraction was assayed against *C. neoformans*, aiming for MIC concentrations close to the one obtained previously (Bresciani et al., 2020). The MIC values for each batch varies between 125 and 312 μg/mL, but all batches used in this work had a MIC of 312 μg/mL (Supplementary Table S4). NMR experiments were also repeated and included as Supplementary Figures S3, S4. Through sequence and structure comparison, chitinase 42 from *Trichoderma harzianum* was chosen as a positive control for chitinolytic activity assays (Supplementary Figure S5). A solution of 1 mg/mL of the Lysing Enzymes from *T. harzianum* (Sigma-Aldrich Co.) was used in each experiment as a control. For *C. neoformans*, chitinolytic activity was significantly higher in the insoluble cell fraction, for cells grown either in YPD or YPGlcNAc, when compared to the other fractions evaluated (Figures 2A,B). For fungal grown in YPD, the chitinolytic activity in the secreted fraction did not show statistical difference when compared to the soluble cell fraction. While fungal grown in YPGlcNAc presented higher chitinolytic activity in the soluble cell fraction than in the secreted fraction (Figures 2A,B).

Secreted and soluble cell fractions of *C. neoformans*, grown in both media, showed a significant reduction in chitinase activity in the presence of plumieridine (Figures 2C,D and Supplementary Figures S6A,B). Noteworthy, the secreted fraction (*C. neoformans* grown in YPD) displayed a constant reduction in the chitinase activity in the presence of plumieridine between 33 and 160 μg/mL (Figure 2C). Similarly, a reduction in chitinolytic activity was also observed (between 33 and 100 μg/mL) for cells grown in YPGlcNAc (Figure 2D). In the soluble cell fraction, a reduction in chitinolytic activity was observed in the assays employing 260 μg/mL of plumieriedine for *C. neoformans* grown in YPD and YPGlcNAc (Supplementary Figures S6A,B).

Conversely, the inhibition of the chitinolytic activity in the insoluble cell fraction did not present the same dose-dependent pattern. For the insoluble cell fraction obtained from cells grown in YPD, the maximum inhibitory activity was observed with 100 μg/mL of plumieridine (Supplementary Figure S6C). While, for YPGlcNAc, a reduction in the chitinolytic activity was observed with 300 μg/mL of plumieridine (Supplementary Figure S6D).

### Transcriptional Levels of CHI22 Are Reduced in the Presence of Plumieridine

**FIGURE 3:**
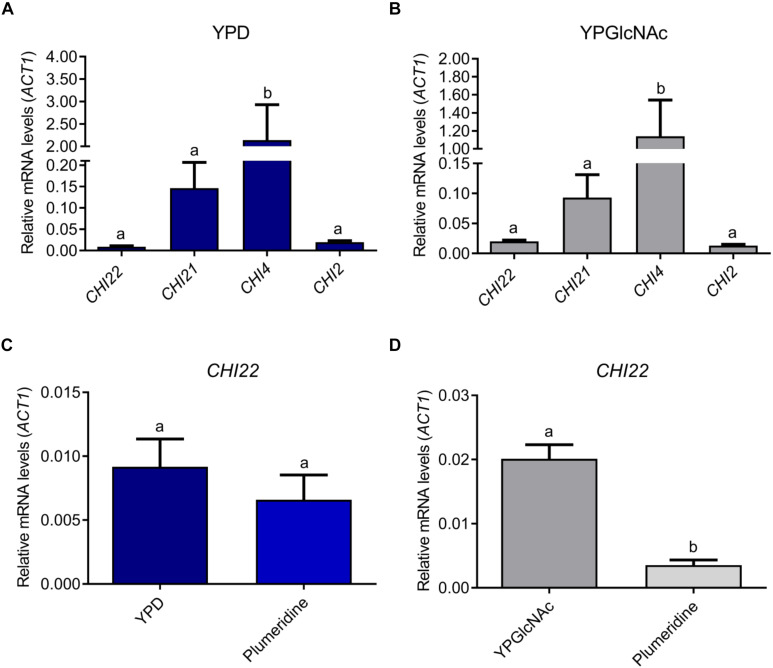
Relative expression levels of *C. neoformans* chitinases. Relative expression levels of *CHI2*, *CHI21*, *CHI22*, and *CHI4* after cryptococcal growth in YPD **(A)** and YPGlcNAc **(B).** *CHI22* expression levels after *C. neoformans* growth in the presence or absence of plumieridine in YPD **(C)** and YPGlcNAc **(D)**. Letters above bars indicate statistical difference among different treatments. The expression data are relative to *ACT1* level.

The qRT-PCR results revealed that the most expressed chitinase gene in *C. neoformans*, independently of the culture media, was *CHI4* (Figures 3A,B). Although *CHI4* showed the highest relative expression levels, it was reported that mutants expressing only *CHI4* or *CHI21* did not present chitinolytic activity (Baker et al., 2009). *CHI2*, *CHI21*, and *CHI4* transcriptional levels were not influenced by the carbon sources tested (glucose or GlcNAc) or treatment with plumieridine (Figures 3A,B and Supplementary Figure S7). Notably, *CHI22* was the only chitinase in which transcriptional levels were influenced by the carbon source evaluated (higher when the yeast was grown in YPGlcNAc). Remarkably, plumieridine treatment negatively affected the expression levels of *CHI22* when the fungus was grown in YPGlcNAc (Figures 3C,D).

### Plumieridine Changes Chitin Oligomers Distribution in *C. neoformans* Cell Wall

**FIGURE 4:**
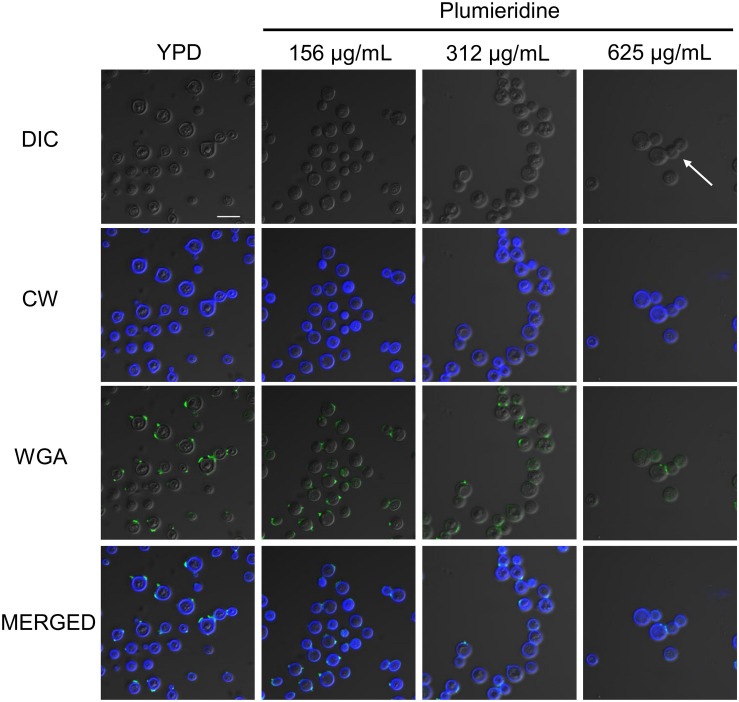
Confocal microscopy of *C. neoformans* treated with different concentrations of the plumieridine-rich fraction. *C. neoformans* was treated with different concentrations of the plumirieidine-rich fraction (0, 156, 312, and 625 μg/mL) and resulting phenotypic alterations were tracked. Differential Interference Contrast (DIC); Calcofluor White (CW; blue); Wheat Germ Agglutinin conjugated with Alexa 488 (WGA; green). Scale bar, 10 μm, for all images.

Bresciani et al. (2020) reported that aqueous extracts of *A. polyantha* seeds induced morphological alterations in *Cryptococcus* spp. The morphological alterations were putatively attributed to plumieridine and plumieride. Thus, we repeated the experiments with the plumieridine-rich fraction to confirm the previous suggestions. Confocal microscopy revealed that plumieridine reduces cell count in a dose-dependent manner (Figure 4). Cell counts of 31, 29, 23, and 7 cells per field were observed in assays employing 0, 156, 312, and 625 μL/mL of plumieridine, respectively. Cells treated with 312 e 625 μL/mL of plumieridine have incomplete mother–daughter separation, evidenced by a group of three cells lined up (Figure 4 and Supplementary Figure S8, white arrow). Changes in chitin from control and treated cells were not observed through calcofluor white staining (Figure 4). However, WGA appears in one or, more frequently, two dots per cell, which can be described as a polarized pattern. Nonetheless, 6.4% (2/31) of the cell count in the control presented a diffuse WGA staining in the cell wall (Figure 4). The diffuse staining pattern increases with higher plumieridine concentrations: 34% (10/29) in the treatment with 156 μg/mL, 43% (10/23) with 312 μg/mL and 57% (4/7) with 625 μg/mL of plumieridine (Figure 4).

### Plumieridine Harbors Fungistatic and Fungicidal Activities

**FIGURE 5:**
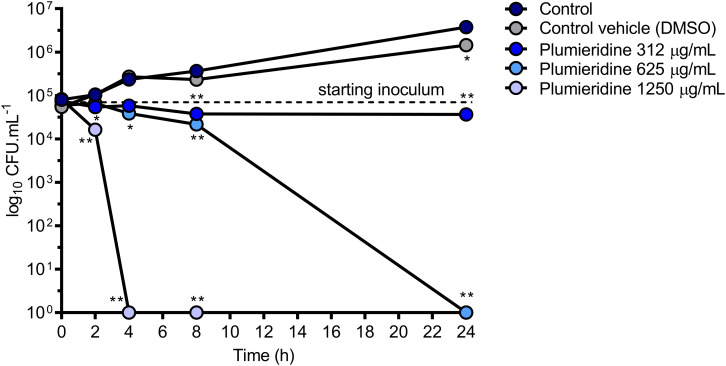
Time to kill assay. *C. neoformans* viability was determined by CFU counting after growth in RPMI 1640 with and without plumieridine supplementation (312, 625, and 1250 μg/mL) at different time points. The experiments were performed in biological and technical duplicates. Water and DMSO 1.25% were employed as control e control vehicle, respectively. *p < 0.05 and **p < 0.01 for comparisons of control results to the other conditions by one-way ANOVA with *post hoc* Dunnet test for each time point.

In the confocal microscopy assay a reduction in the fungal loads was observed, which pointed for a potential fungicidal activity of the plumieridine-rich fraction. To evaluate the potential fungistatic and fungicidal activities of plumieridine treatment in *C. neoformans*, a time kill assay was employed and the reduction in the fungal loads was followed through CFU counting (Figure 5). When *C. neoformans* was treated with the MIC concentration, a fungistatic activity was observed with minimal fluctuation in the fungal loads for up to 24 h (Figure 5). On the other hand, a fungicidal activity was observed in the treatments employing 625 and 1250 μg/mL (Figure 5).

### Plumieridine Exerts Inhibitory Activity Against Several Chitinases From GH18 Superfamily

**FIGURE 6:**
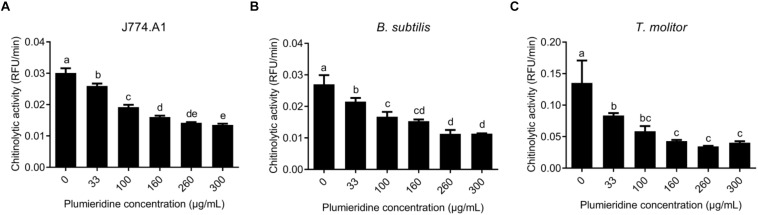
Inhibition of chitinolytic activity by plumieridine in different models. **(A)** Inhibition of chitinolytic activity in the supernatant obtained from the culture of mouse-derived macrophage cells (lineage J774.A1); **(B)** Inhibition of chitinolytic activity in the supernatant obtained from the culture of *B. subtilis* ATCC6633; **(C)** Inhibition of chitinolytic activity in the filtered crude extract of the mealworm larvae *T. molitor*. Letters above bars indicate statistical difference among different treatments.

The local alignment showed that human chitinase (PDB 1HKI) (Rao et al., 2003) and mouse chitinase (PDB 1VF8) (Tsai et al., 2004) possess 48% of identity and 67% of similarity (data not shown). Structure comparison reveals that these chitinase structures present a superposition of 0.692 Å (Supplementary Figure S9). Based on this similarity, the chitinases from mouse-derived macrophage cells (J774.A1) were employed as a model in the assays of chitinolytic inhibitory activity, and these results can potentially be applied for humans. Chitinase inhibitory assays employing J774.A1 supernatant revealed a constant reduction in the chitinase activity after treatment with plumieridine (between 33 and 260 μg/mL), whereas higher plumieridine concentrations failed to reduce the chitinolytic activity even more (Figure 6A). Similar results were found employing *B. subtilis* ATCC6633 supernatant (Figure 6B), where chitinolytic activity was inhibited up to 260 μg/mL, with no further enhancement with a higher amount of plumieridine. *T. molitor* supernatant showed significant inhibition of chitinase activity in treatment employing the plumieridine concentrations up to 160 μg/mL with no further enhancement with higher concentrations of the compound (Figure 6C). Given the promiscuous inhibitory activity presented by plumieridine on different organisms (all harboring GH18 chitinases) and that the simulations showed the interaction of the compound with catalytic residues of chitinases from several sources, our results suggest that plumieridine can be, potentially, a broad-spectrum GH18 superfamily inhibitor.

### Plumieridine Interacts Differently With *C. neoformans* Chitinases

**FIGURE 7:**
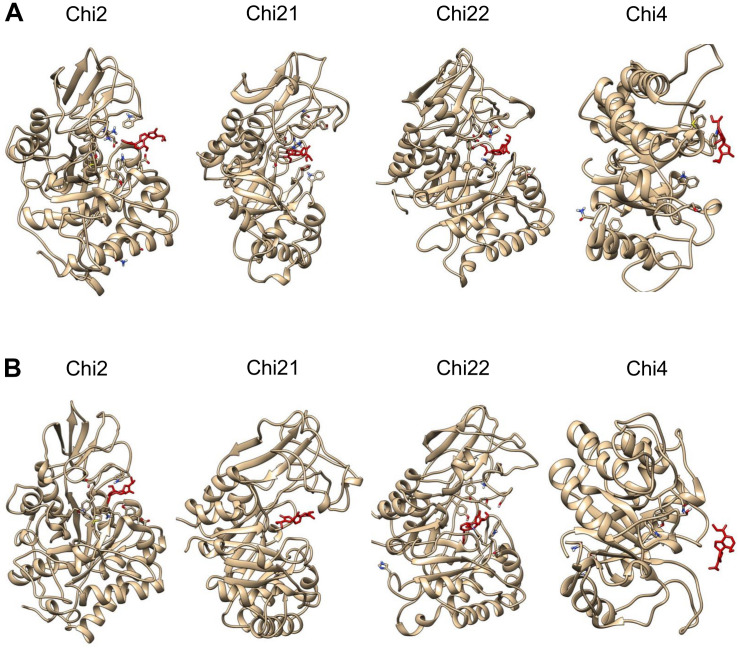
The interaction of the complexes chitinases-plumieridine after dynamics simulation with initial inward/outward ligand’s orientation. Panels with inward **(A)** and outward **(B)** plumieridine orientations regarding the active site of each *C. neoformans* chitinases. Plumieridine (red).

All plumieridine-chitinase complexes reached system equilibrium evidenced by RMSD analysis (Supplementary Figure S10). Molecular dynamics reveals that the inward plumieridine orientation is the most energetically favorable binding configuration (Supplementary Figure S11). For Chi2-, Chi21-, and Chi22-plumieridine complexes, in the initial outward orientation, we observed that plumieridine rotates and changes to the inward orientation at the end of the simulation (Figure 7 and Supplementary Figure S12). This suggests that plumieridine, only in the inward orientation, can inhibit the activity of *C. neoformans* chitinases.

Controversially, Chi4-plumieridine complexes in both orientations had the inhibitor expelled from the active site. This suggests that plumieridine is prone to inhibit GH18 superfamily chitinases, however, interactions potentially happen according to affinities for residues in the active site (Figure 7 and Supplementary Figure S12). It also suggests that plumieridine inhibits GH18 chitinases selectively and in different levels, thus, these results can also explain the residual chitinase activity observed in the assays employing the higher concentrations of the compound (Figures 2C,D, 5 and Supplementary Figure S6).

### *In silico* Toxicity Assay

To explore the potential toxicity of the plumieiridine in rats, an *in silico* approach was employed. The prediction pointed to an LD_50_ value of 79,04 mg/kg (Supplementary Table S5), which was based on similarities between plumieridine and known compounds in the databases (Supplementary Figure S13). According to the Hodge and Sterner Scale of toxicity classes, plumieridine can be classified as moderately toxic (Hodge and Sterner, 1949; Erhirhie et al., 2018). The compounds are classified as moderately toxic when the oral LD_50_ ranges between 50–500 mg/kg (single dose to rats) (Hodge and Sterner, 1949).

## Discussion

There is a constant need for new, cheaper, less toxic, and widely available drugs for cryptococcosis treatment (Coelho and Casadevall, 2016; Mourad and Perfect, 2018a, b). In this way, South America’s biodiversity can be a rich source of new molecules (Valli et al., 2012). The antifungal activity displayed by plumieridine against *C. neoformans*, led us to investigate the potential drug-target interactions. The time from the discovery and trials of a potential drug is estimated to be around 14 years (Song et al., 2009) and costs US$ 800 million (Lavecchia and Di Giovanni, 2013). Additionally, detailed information about drug-target interactions can also consume several years. Virtual screening approaches (as those applied here to identify chitinases as potential plumieridine targets) can reduce substantially the research time, providing detailed information on drug-target interactions (Kitchen et al., 2004). To support the results found through the virtual screening approaches several chitinolytic assays were conducted. Remarkably, relative chitinolytic activity in *C. neoformans*-soluble fractions was reduced by the presence of plumieridine, proving the efficiency of our approach.

Although previous reports have pointed that chitinases are not required for asexual reproduction in *C. neoformans KN99a* and *KN99*α (Baker et al., 2009), our results suggest that partial impairment of chitinolytic activity can lead to reductions in the cell count due to asexual reproduction impairment, as well as to other potential morphological alterations. When chitinase activity is absent due to plumieridine interaction, cell aggregation and incomplete cytokinesis can be observed. Moreover, the MIC concentration (325 μg/mL) displayed fungistatic activity, while the treatment with 625 or 1250 μg/mL of the plumieridine-rich fraction exerted fungicidal activity. Notably, treatment of *C. neoformans* with other chitinase inhibitors (methylxanthines) resulted in phenotypes similar to the ones observed for the treatments with the aqueous extracts of *A. polyantha* or the plumieridine-rich fraction (Tsirilakis et al., 2012; Bresciani et al., 2020).

Even though the molecular docking experiments have predicted that plumieridine would be effective against all *C. neoformans* chitinases, chitinolytic activity in the insoluble fraction was not constant with increasing plumieridine concentrations. As predicted by TMHMM, Chi2 possesses a transmembrane helix and may be responsible for chitinolytic activity detected in the insoluble cell fraction. This suggests that Chi2 activity might not be inhibited by plumieridine. Additionally, molecular dynamics simulations indicated that Chi4 may not interact with plumieridine in the same way that other *C. neoformans* chitinases interact. The weaker or lack of affinity observed in the simulations suggests that plumieridine can have specificity for some residues in the active site of chitinases.

Allosamidin is a known chitinase inhibitor isolated from *Streptomyces* spp. and this compound can also regulate chitinase production in these bacteria. Besides regulating chitinase production, allosamidin does not inhibit the chitin-hydrolytic activity of *Streptomyces* chitinases (Suzuki et al., 2006). On the other hand, plumieridine reduced *C. neoformans* chitinolytic activity but affected only the *CHI22* expression levels. These results suggest that plumieridine is not involved in cryptococcal chitinase transcription regulation.

The virtual screening approach employed here to identify potential targets of plumieridine pointed to the complex chitinase B1- tripeptide VR0 (PDB ID 3CHE) (i.e., VR0 is derived from the chitinase inhibitor argifin) (Andersen et al., 2008). Structural analyses revealed that the interactions in *C. neoformans* chitinase-plumieridine complexes occur with residues conserved in the chitinase catalytic motif (DxDxE) or in the vicinities of the conserved motif (Supplementary Figure S12). The initial position of plumieridine was the same as the VR0 in *A. fumigatus* chitinase, however, after dynamic simulations, the inhibitor’s position varied in the active site. Although, interactions between the catalytic motif and plumieridine were still observed in Chi2, Chi21, and Chi22 (Supplementary Figure S12). Thus, plumieridine binding is similar to argifin (i.e., a general GH18 superfamily inhibitor) (Andersen et al., 2008) suggesting a conserved inhibitory activity behavior. To test this hypothesis, *in vitro* and *in silico* experiments were conducted with supernatant and cell-free extracts of bacteria, insect and mouse-derived macrophage cells. Inhibition of chitinolytic activity was observed in these assays, further supporting plumieridine as a broad-spectrum chitinase inhibitor and pointing for applications beyond cryptococcosis treatment. In this way, numerous chitinase inhibitors have been reported with several applications (Rao et al., 2005a; Schüttelkopf et al., 2010; Chen et al., 2014; Christy and Jayaprakash, 2017). The already described argifin has an insecticide potential demonstrated in *Periplaneta americana* (Omura et al., 2000). Furthermore, chitinase inhibitors can also be employed to relieve the symptoms of respiratory diseases, since chitinolytic activity and chitinase expression levels increases during pulmonary inflammations, aggravating it (Létuvé et al., 2010; Mazur et al., 2019). For instance, the already described allosamidin was observed to reduce the asthma inflammatory process by reducing lymphocyte and eosinophil recruitment to mouse lungs (Zhu et al., 2004).

Finally, to explore the potential toxicity of plumieridine, an *in silico* oral toxicity assay was employed. According to TEST prediction, plumieridine is moderately toxic. The *in silico* approach adds up with fibroblast viability assay conducted previously, employing aqueous extracts of *A. polyantha* (Bresciani et al., 2020). For the aqueous extracts, concentrations lower than 70 μg/mL did not affect cell viability, while 563 μg/mL (*C. neoformans* strain H99 crude extract MIC concentration) reduced cell viability around 82% (Bresciani et al., 2020). However, the toxicity of the pure plumieridine still needs further investigation with cytotoxic assays, to correctly set the potential applications of this compound.

## Data Availability Statement

All datasets generated for this study are included in the article/Supplementary Material.

## Author Contributions

MV, JL, EB, ES, JR, and NS contributed for experimental design and results interpretation. ES, VB, and NS contributed with seed extraction, MIC assays, and chromatographic fractionation. ES and JR performed the qRT-PCR. AS contributed with time to kill assay. ES and RS contributed with chitinolytic activity assays. GP contributed with NMR analysis. AG contributed with macrophages cell culture and supernatant extraction. NS and ES contributed with *T. molitor*’s assay. ES, JR, and NS contributed with manuscript redaction. All the authors contributed to the article and approved the submitted version.

## Conflict of Interest

The authors declare that the research was conducted in the absence of any commercial or financial relationships that could be construed as a potential conflict of interest.
