# Supplementary material for: A Plumieridine-Rich Fraction From Allamanda polyantha Inhibits Chitinolytic Activity and Exhibits Antifungal Properties Against Cryptococcus neoformans
Source: Front Microbiol. 2020 Aug 28;11:2058. doi: 10.3389/fmicb.2020.02058 (PMC7483551; doi:10.3389/fmicb.2020.02058)
Supplement: Supplementary file 1 [file Table_1.DOCX]

***Supplementary Material 1***

**Table S1. Primers used in this study.**

| **Gene** | **Primer name** | **Purpose** | **Sequence (5’-3’)** |
| --- | --- | --- | --- |
| *CHI2* | CNAG_03412 F | Amplify *CHI2* for RT-qPCR | GAAGCATCTCCTCATTACCTATG |
| *CHI2* | CNAG_03412 R | Amplify *CHI2* for RT-qPCR | CTCACAGTCCTCACCAATGAC |
| *CHI21* | CNAG_02598 F | Amplify *CHI21* for RT-qPCR | CCGACGGAAGTACAGAGATTA |
| *CHI21* | CNAG_02598 R | Amplify *CHI21* for RT-qPCR | GTTGAGACTCCAGACCATAGC |
| *CHI22* | CNAG_04245 F | Amplify *CHI22* for RT-qPCR | CTCCAGCCGTTCTTTCATTTG |
| *CHI22* | CNAG_04245 R | Amplify *CHI22* for RT-qPCR | CAGTGGTGGTAGCGGTAATAG |
| *CHI4* | CNAG_02351 F | Amplify *CHI4* for RT-qPCR | GTTCTCTGAACTCGACTCTTT |
| *CHI4* | CNAG_02351 R | Amplify *CHI4* for RT-qPCR | TAGACCCAAAGGCAGCAATC |
| *ACT1* | CNAG_00483 F | Amplify *ACT1* for RT-qPCR | CAAGCAGAACCGAGAGAAG |
| *ACT1* | CNAG_00483 R | Amplify *ACT1* for RT-qPCR | CGTCACCAGAGTCAAGAAC |

**Table S2. List of predicted plumieridine targets based on *A. fumigatus* protein structures.** Virtual screening was performed through a ligand-based approach using pharmACOphore to compare the similarities between ligands. The results were filtered for *A. fumigatus* proteins structures using the Protein Data Base (PDB) and each ligand was complexed in the identified structures.

| **Protein** | **PDB ID** | **Ligand** |
| --- | --- | --- |
| Chitinase | 3CHD | Dipeptide (WRG) |
| Farnesyltransferase | 4LNG | Farnesyldiphosphate and tipifarnib |
| Chitinase | 2A3C | Pentoxifylline |
| Farnesyltransferase | 4LNB | Farnesyldiphosphate and ethylenediamine |
| Chitinase | 1W9V | Cyclopentapeptide (argifin) |
| Chitinase | 2IUZ | C2-dicaffeine |
| Chitinase | 3CHC | Monopeptide (ZRG) |
| Chitinase | 4TX6 | 3-(2-methoxyphenyl)-6-methyl[1,2]oxazolo[5,4-d]  pyrimidin-4(5H)-one |
| Chitinase | 2XTK | Acetazolamide |
| Chitinase | 3CHE | Tripeptide (VR0) |
| Chitinase | 3CHF | Tetrapeptide (VR0) |
| Chitinase | 2A3A | Theophylline |
| Chitinase | 3CH9 | Dimethylguanylurea |
| Fucose binding lectin | 4D52 | L-Galactopyranose |
| FtmOx1 | 4Y5S | 2-Oxoglutaric acid |
| Chitinase | 2A3B | Caffeine |
| Chitinase | 2XUC | 1-methyl-3-(N-methylcarbamimidoyl)urea |
| Chitinase | 1W9U | Cyclopentapeptide (argadin) |
| Fucose binding lectin | 4C1Y | B-methylfucoside |

**Table S3. *C. neoformans* chitinases.** Prediction of signal peptide (SignalP), transmembrane helices (TMHMM) and conserved domains (Conserved Domain Database) for each *C. neoformans* serotype A chitinase. N-terminal sequence was retrieved from Baker and collaborators (2009).

| **Chitinase** | **FungiDB ID** | | **Transmembrane helix** | **Conserved domain** | **Signal peptide** | **N-terminal sequence** |
| --- | --- | --- | --- | --- | --- | --- |
| Chi2 | | CNAG_03412 | Yes | GH18 chitinase like (cl10447) | No | None |
| Chi21 | | CNAG_02598 | No | Glyco hydro 18 (cl23725) | Yes | MHFVGSTTLFVILTALAVRSA |
| Chi22 | | CNAG_04245 | No | Glyco hydro 18 (cl23725) | Yes | MFLSTPAVLSFVLLLASQSSAQ |
| Chi4 | | CNAG_02351 | No | GH18 chitinase like (cl10447) | Yes | MYCTLATLSLLALAEA |

Adapted from (Baker et al., 2009).

**Table S4. Minimal Inhibitory Concentration (MIC) values of the plumieridine-rich fraction extracted from *A. polyantha* seeds against *C. neoformans*.** Fluconazole and Amphotericin B were used as control.

|  |  | **Plumieridine (µg/mL)** | **Fluconazole (µg/mL)** | **Amphoterin B (µg/mL)** |
| --- | --- | --- | --- | --- |
| **Species** | **Strain** |  |  |  |
| *Cryptococcus neoformans* | H99 | 312 | 64 | 0.0625 |

**Table S5. TEST prediction results for plumieridine.**


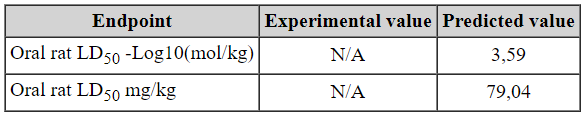


**References**

Baker, L. G., Specht, C. A., and Lodge, J. K. (2009). Chitinases are essential for sexual development but not vegetative growth in Cryptococcus neoformans. *Eukaryot. Cell* 8, 1692–1705. doi:10.1128/EC.00227-09.
