# Supplementary material for: A Plumieridine-Rich Fraction From Allamanda polyantha Inhibits Chitinolytic Activity and Exhibits Antifungal Properties Against Cryptococcus neoformans
Source: Front Microbiol. 2020 Aug 28;11:2058. doi: 10.3389/fmicb.2020.02058 (PMC7483551; doi:10.3389/fmicb.2020.02058)
Supplement: Supplementary file 2 [file Presentation_1.PPTX]

## Slide 1
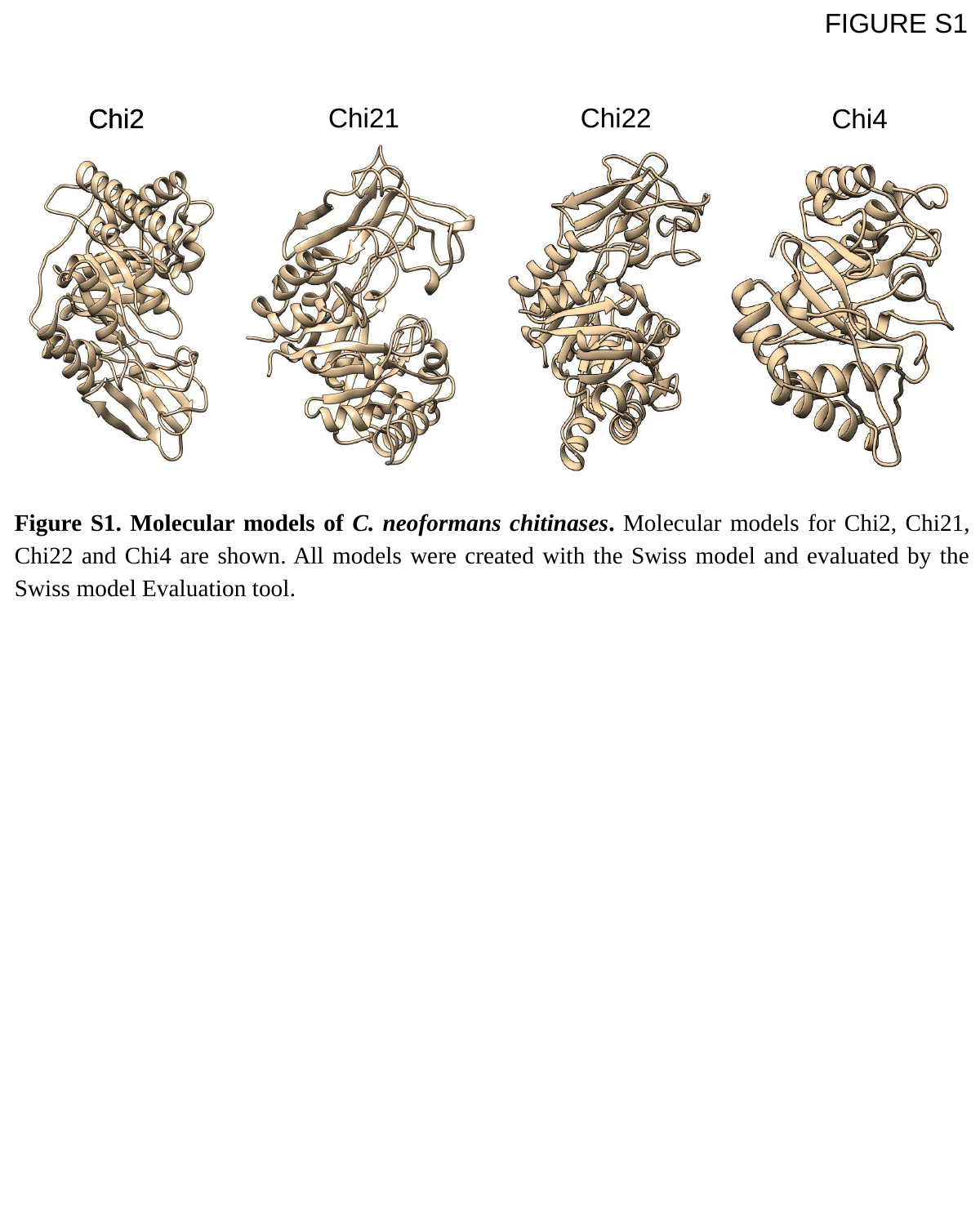

FIGURE S1
Chi21
Chi22
Chi4
Chi2
Chi2
Figure S1. Molecular models of C. neoformans chitinases. Molecular models for Chi2, Chi21, Chi22 and Chi4 are shown. All models were created with the Swiss model and evaluated by the Swiss model Evaluation tool.

## Slide 2
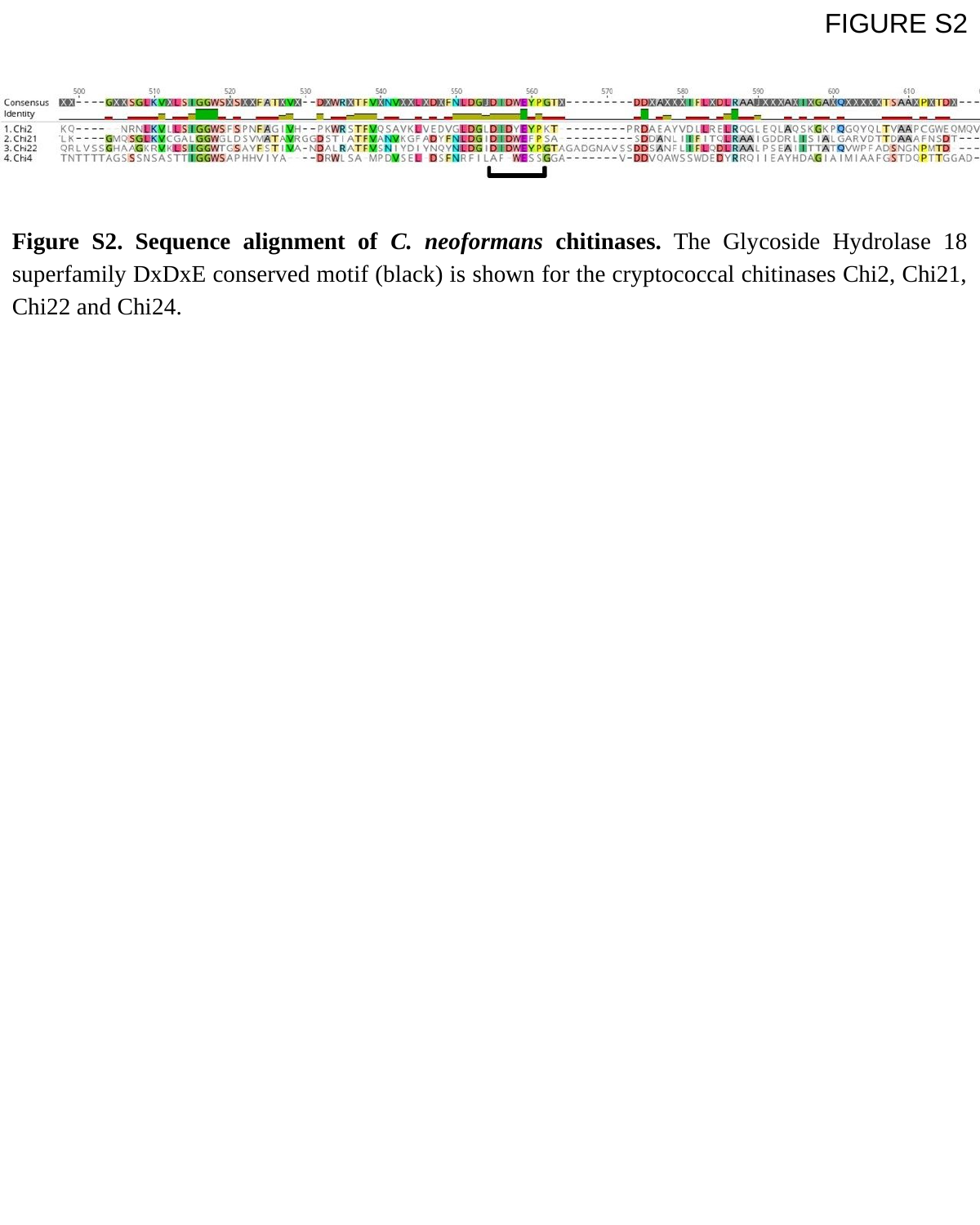

FIGURE S2
Figure S2. Sequence alignment of C. neoformans chitinases. The Glycoside Hydrolase 18 superfamily DxDxE conserved motif (black) is shown for the cryptococcal chitinases Chi2, Chi21, Chi22 and Chi24.

## Slide 3
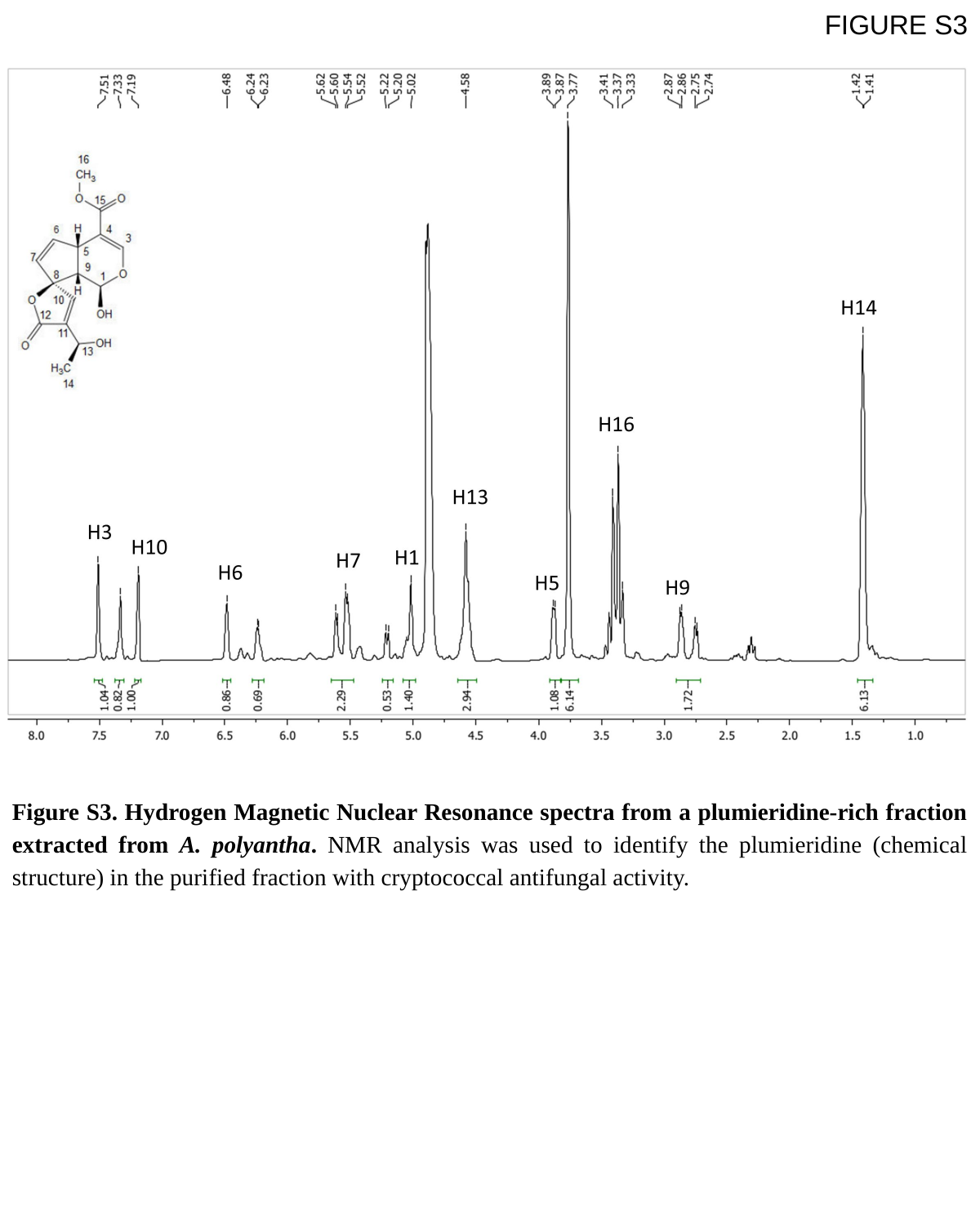

FIGURE S3
Figure S3. Hydrogen Magnetic Nuclear Resonance spectra from a plumieridine-rich fraction extracted from A. polyantha. NMR analysis was used to identify the plumieridine (chemical structure) in the purified fraction with cryptococcal antifungal activity.

## Slide 4
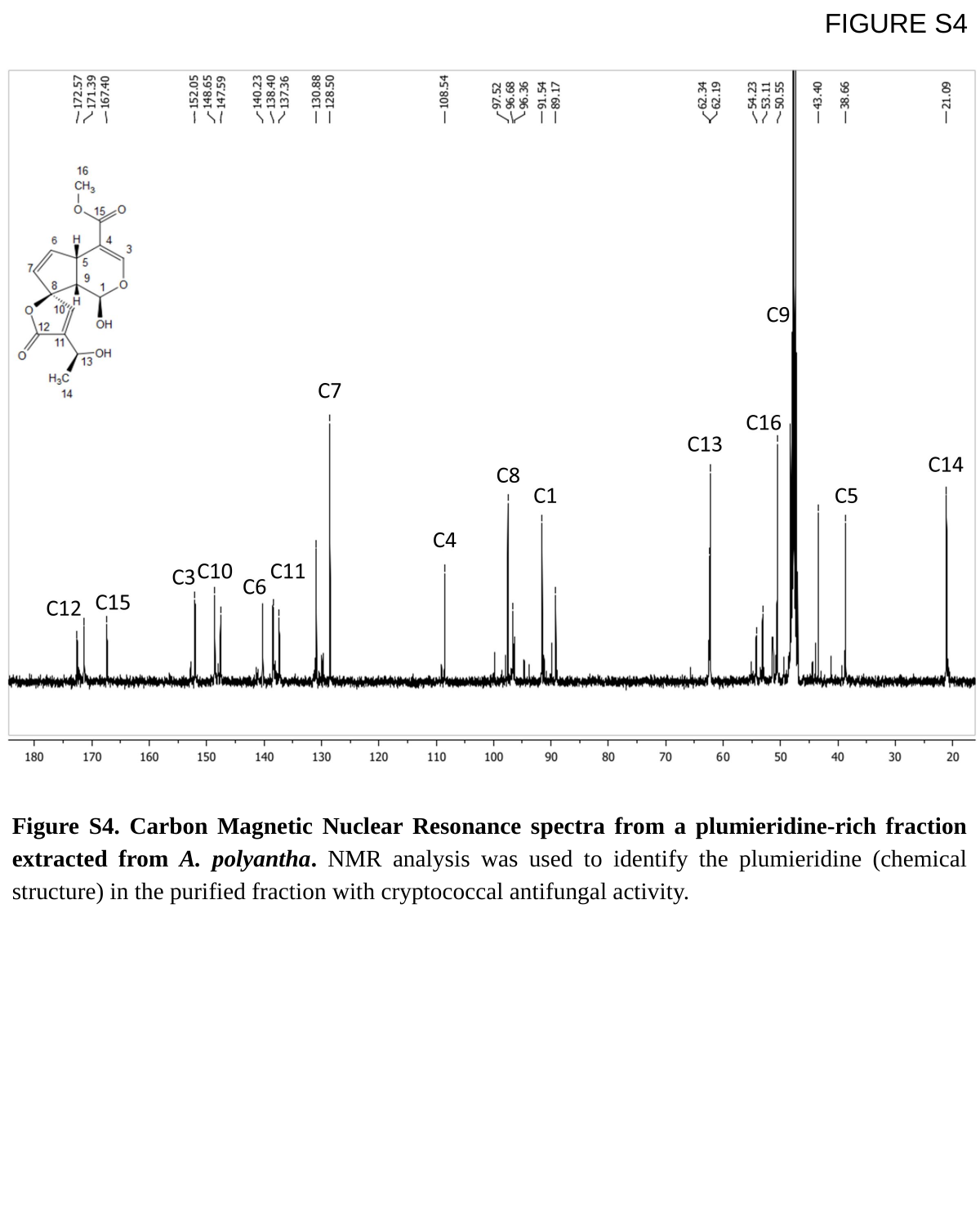

FIGURE S4
Figure S4. Carbon Magnetic Nuclear Resonance spectra from a plumieridine-rich fraction extracted from A. polyantha. NMR analysis was used to identify the plumieridine (chemical structure) in the purified fraction with cryptococcal antifungal activity.

## Slide 5
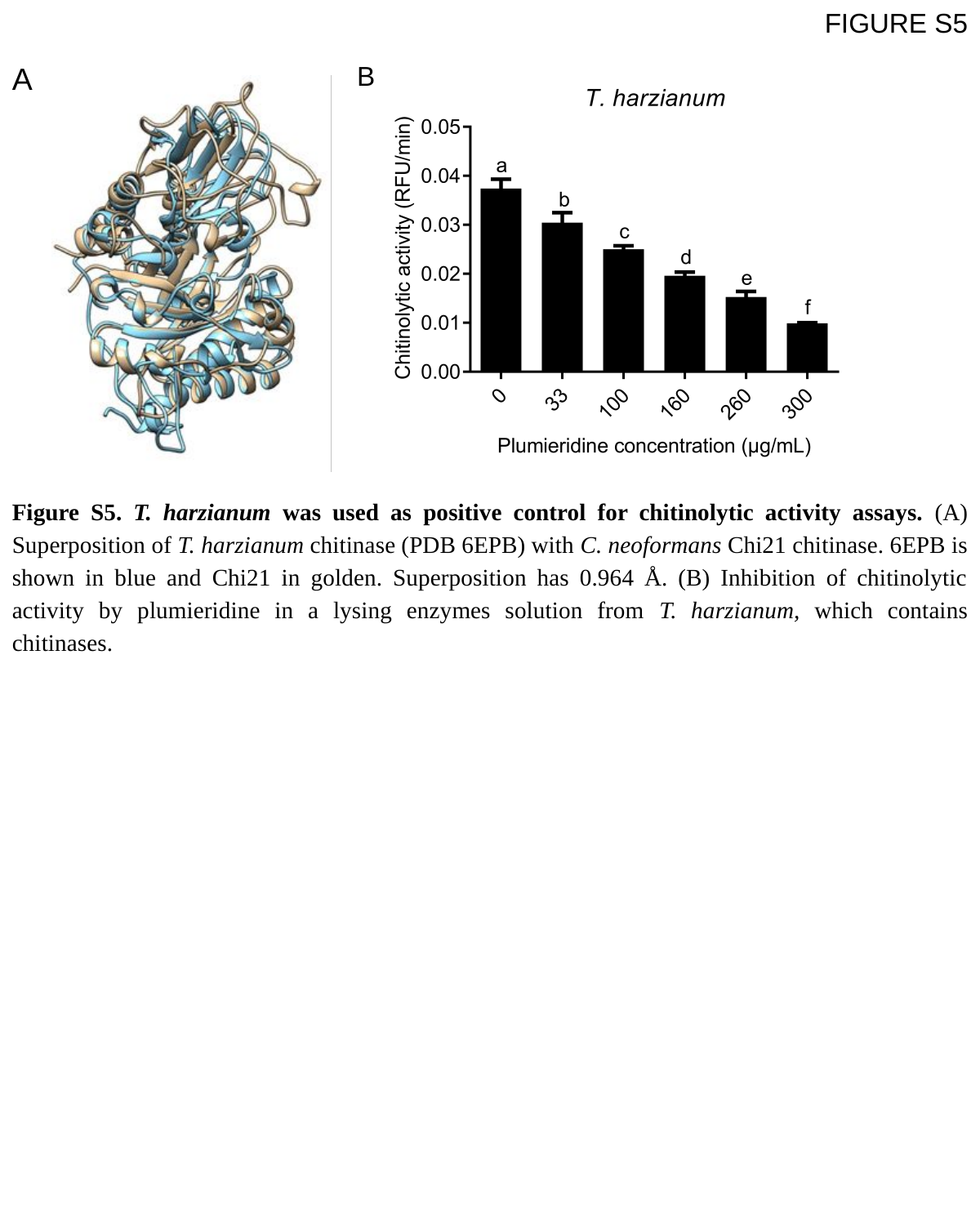

FIGURE S5
B
A
Figure S5. T. harzianum was used as positive control for chitinolytic activity assays. (A) Superposition of T. harzianum chitinase (PDB 6EPB) with C. neoformans Chi21 chitinase. 6EPB is shown in blue and Chi21 in golden. Superposition has 0.964 Å. (B) Inhibition of chitinolytic activity by plumieridine in a lysing enzymes solution from T. harzianum, which contains chitinases.

## Slide 6
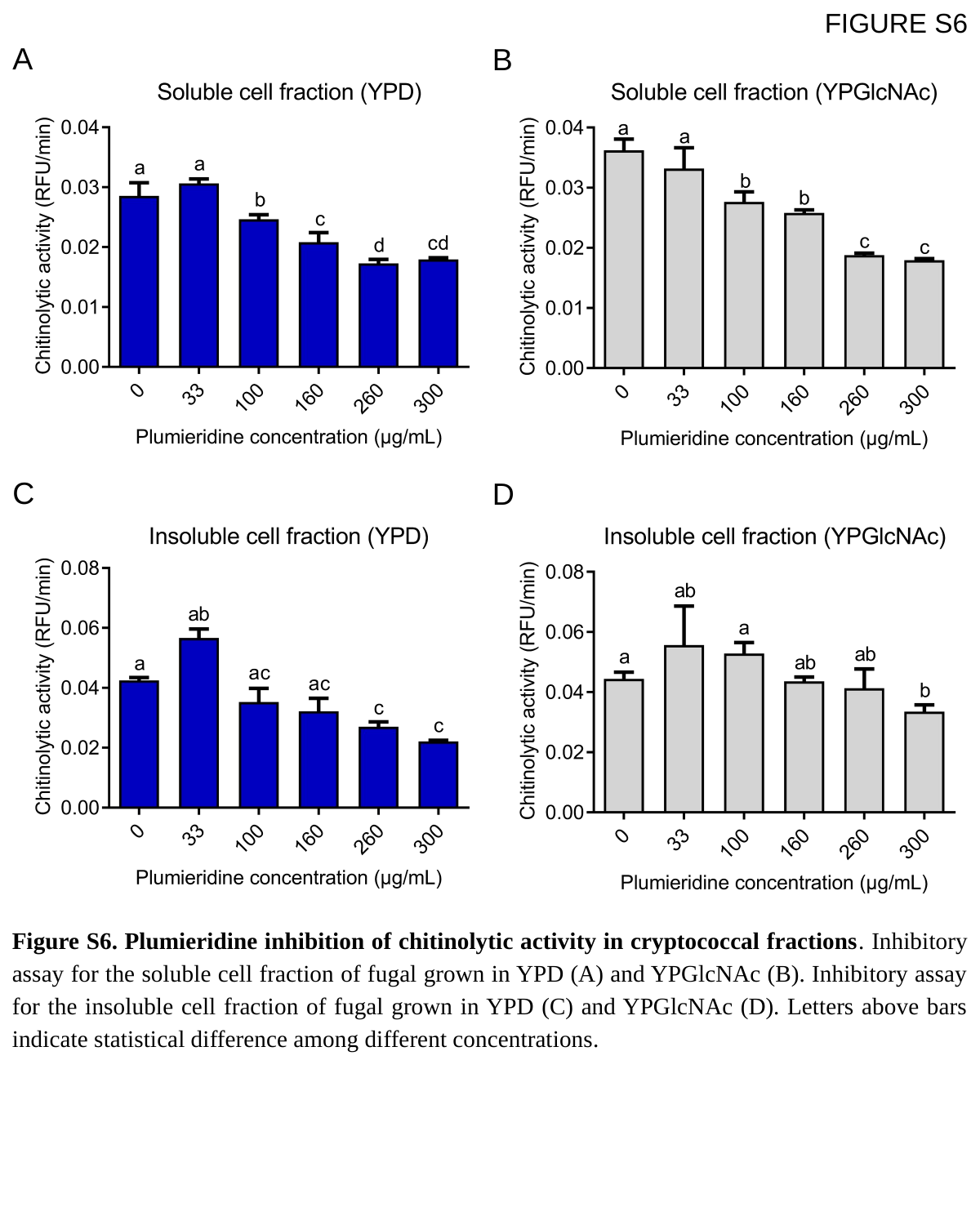

FIGURE S6
A
B
C
D
Figure S6. Plumieridine inhibition of chitinolytic activity in cryptococcal fractions. Inhibitory assay for the soluble cell fraction of fugal grown in YPD (A) and YPGlcNAc (B). Inhibitory assay for the insoluble cell fraction of fugal grown in YPD (C) and YPGlcNAc (D). Letters above bars indicate statistical difference among different concentrations.

## Slide 7
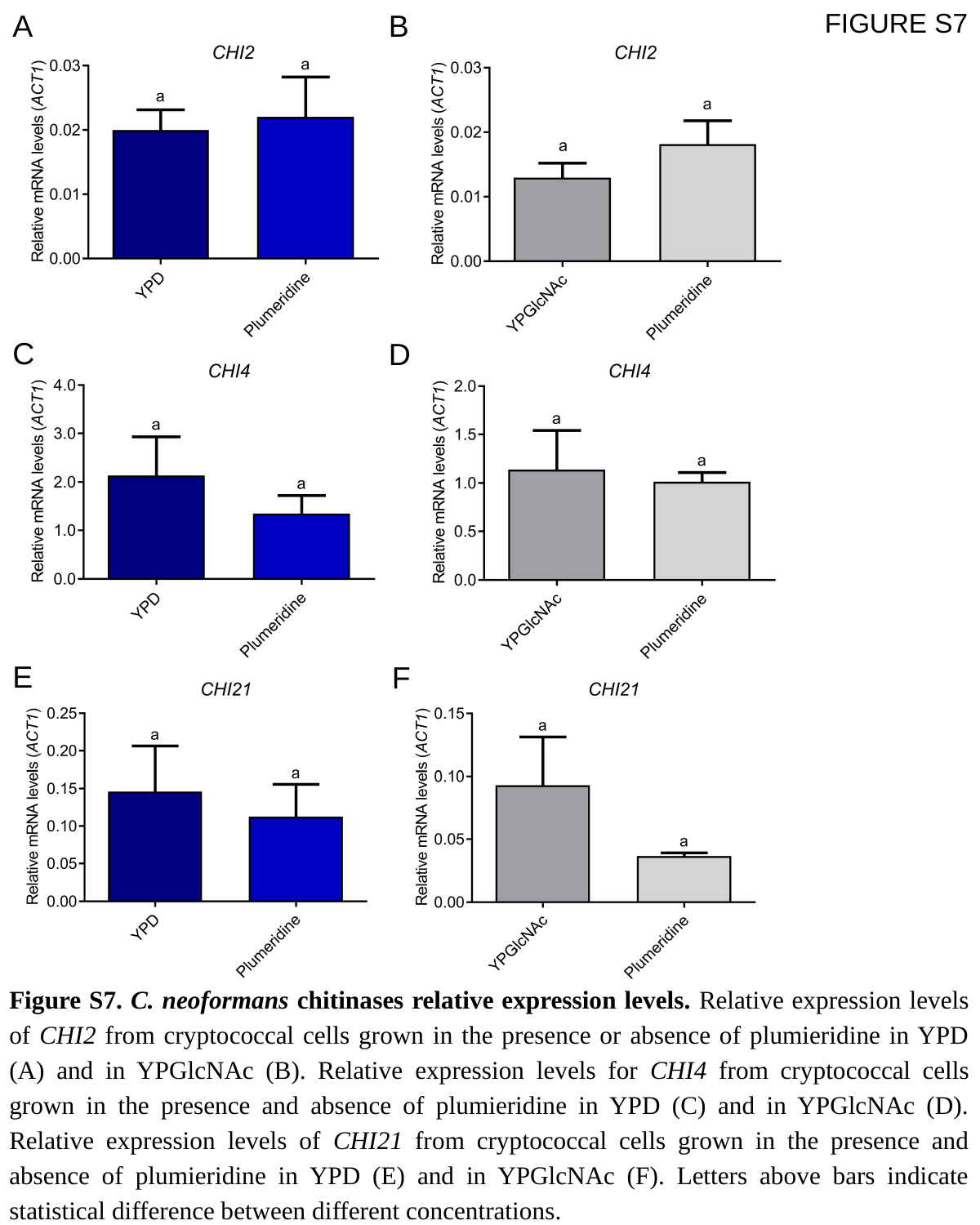

FIGURE S7
A
B
C
D
E
F
Figure S7. C. neoformans chitinases relative expression levels. Relative expression levels of CHI2 from cryptococcal cells grown in the presence or absence of plumieridine in YPD (A) and in YPGlcNAc (B). Relative expression levels for CHI4 from cryptococcal cells grown in the presence and absence of plumieridine in YPD (C) and in YPGlcNAc (D). Relative expression levels of CHI21 from cryptococcal cells grown in the presence and absence of plumieridine in YPD (E) and in YPGlcNAc (F). Letters above bars indicate statistical difference between different concentrations.

## Slide 8
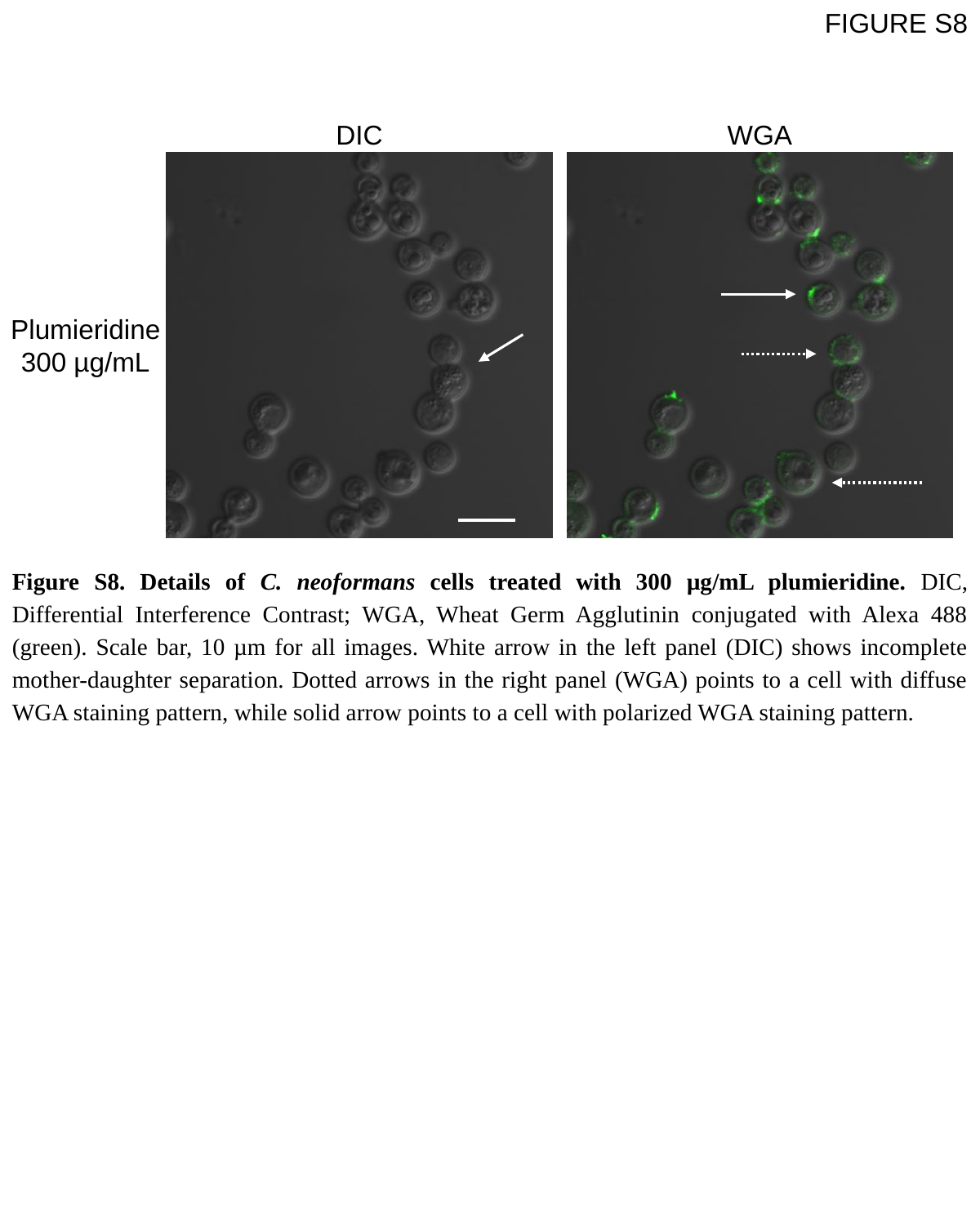

FIGURE S8
DIC
WGA
Plumieridine
300 µg/mL
Figure S8. Details of C. neoformans cells treated with 300 µg/mL plumieridine. DIC, Differential Interference Contrast; WGA, Wheat Germ Agglutinin conjugated with Alexa 488 (green). Scale bar, 10 µm for all images. White arrow in the left panel (DIC) shows incomplete mother-daughter separation. Dotted arrows in the right panel (WGA) points to a cell with diffuse WGA staining pattern, while solid arrow points to a cell with polarized WGA staining pattern.

## Slide 9
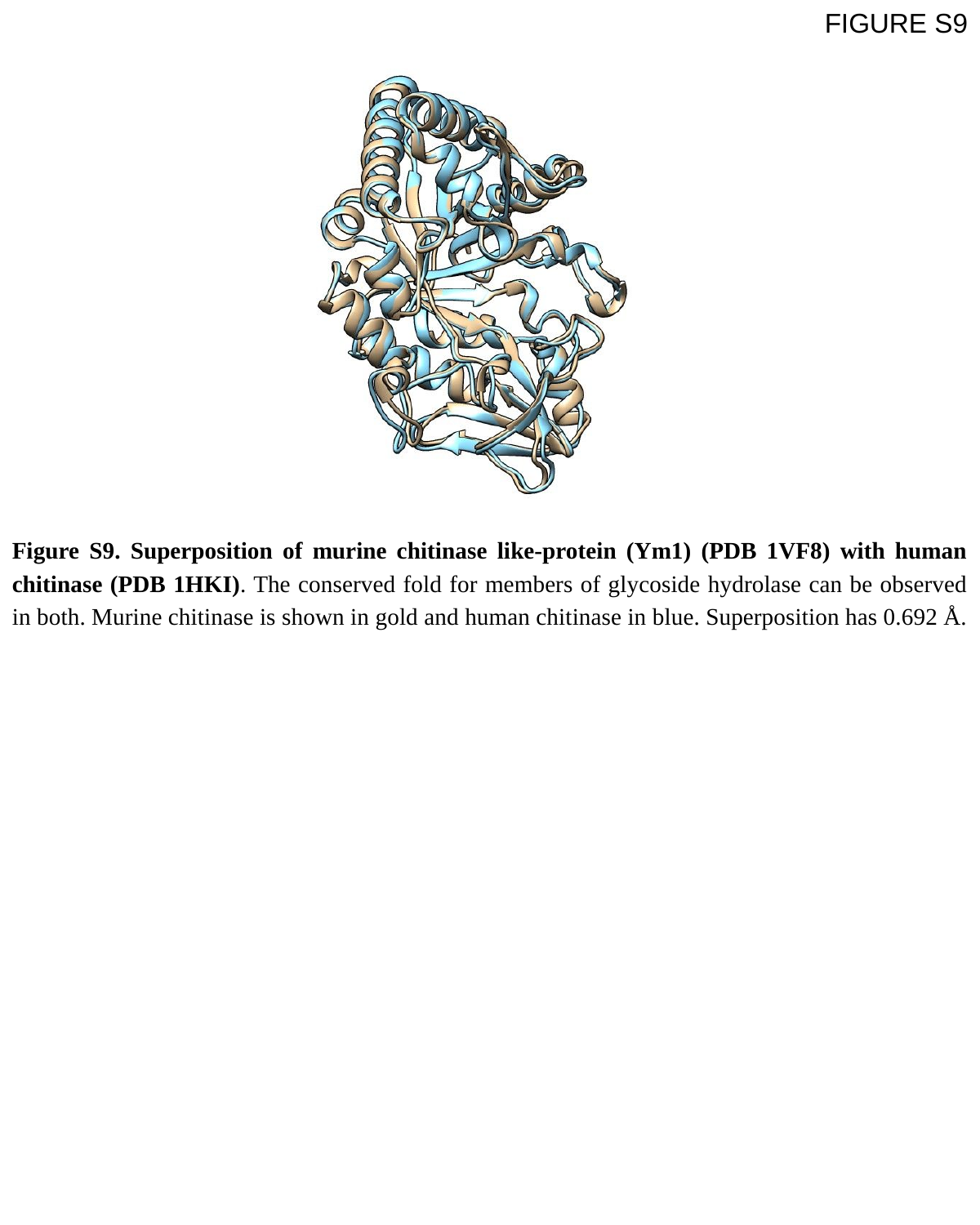

FIGURE S9
Figure S9. Superposition of murine chitinase like-protein (Ym1) (PDB 1VF8) with human chitinase (PDB 1HKI). The conserved fold for members of glycoside hydrolase can be observed in both. Murine chitinase is shown in gold and human chitinase in blue. Superposition has 0.692 Å.

## Slide 10
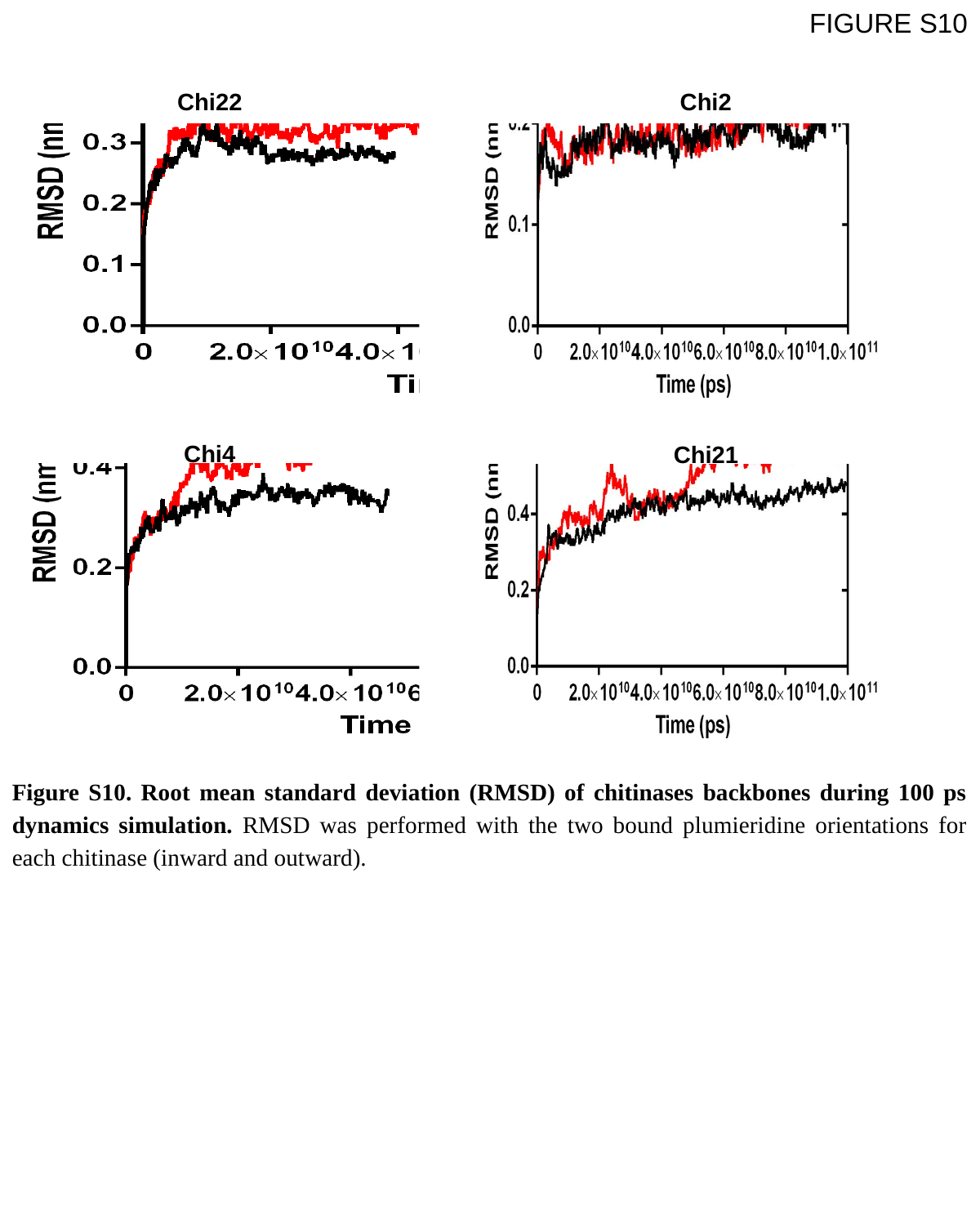

FIGURE S10
Chi22
Chi2
Chi4
Chi21
Figure S10. Root mean standard deviation (RMSD) of chitinases backbones during 100 ps dynamics simulation. RMSD was performed with the two bound plumieridine orientations for each chitinase (inward and outward).

## Slide 11
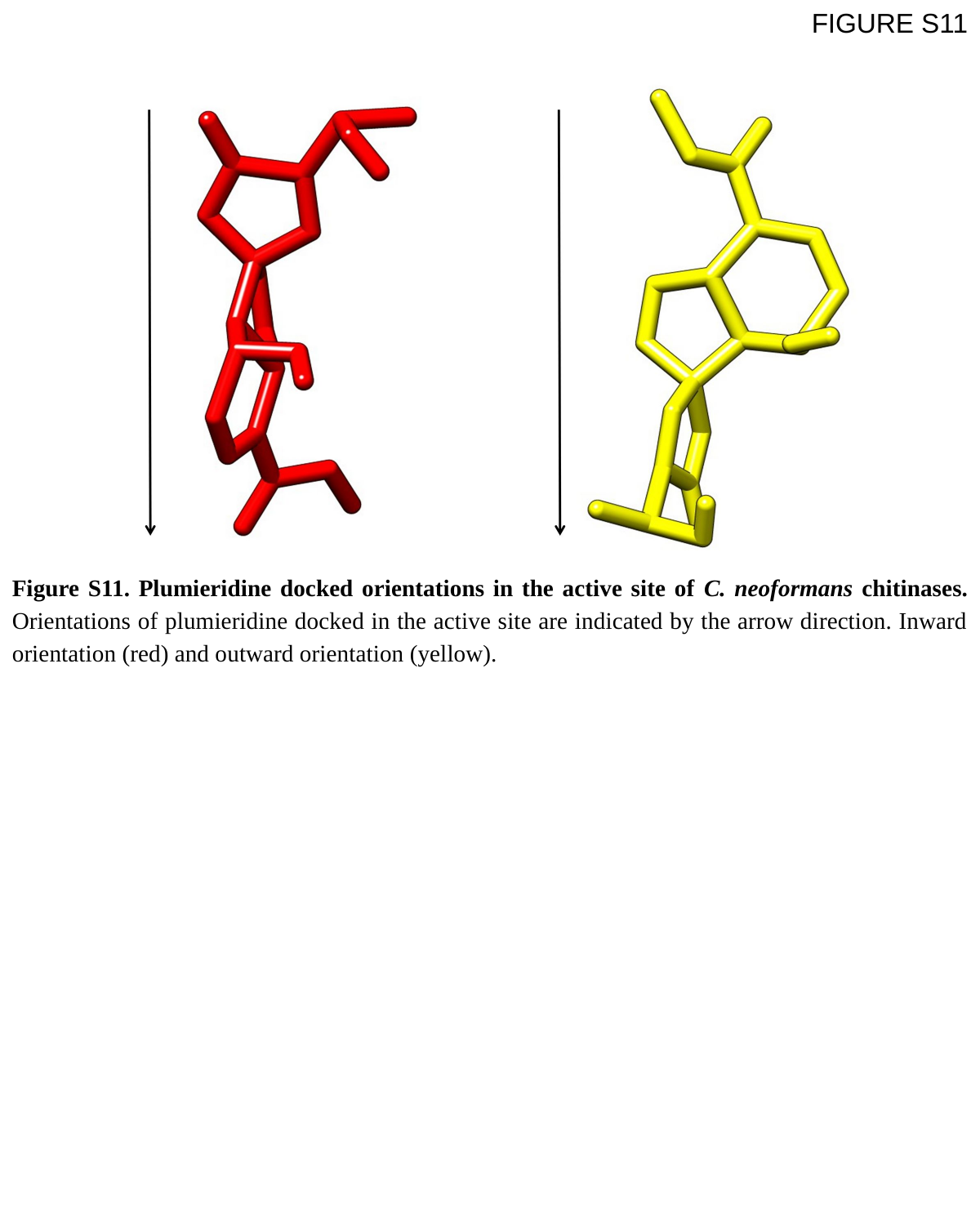

FIGURE S11
Figure S11. Plumieridine docked orientations in the active site of C. neoformans chitinases. Orientations of plumieridine docked in the active site are indicated by the arrow direction. Inward orientation (red) and outward orientation (yellow).

## Slide 12
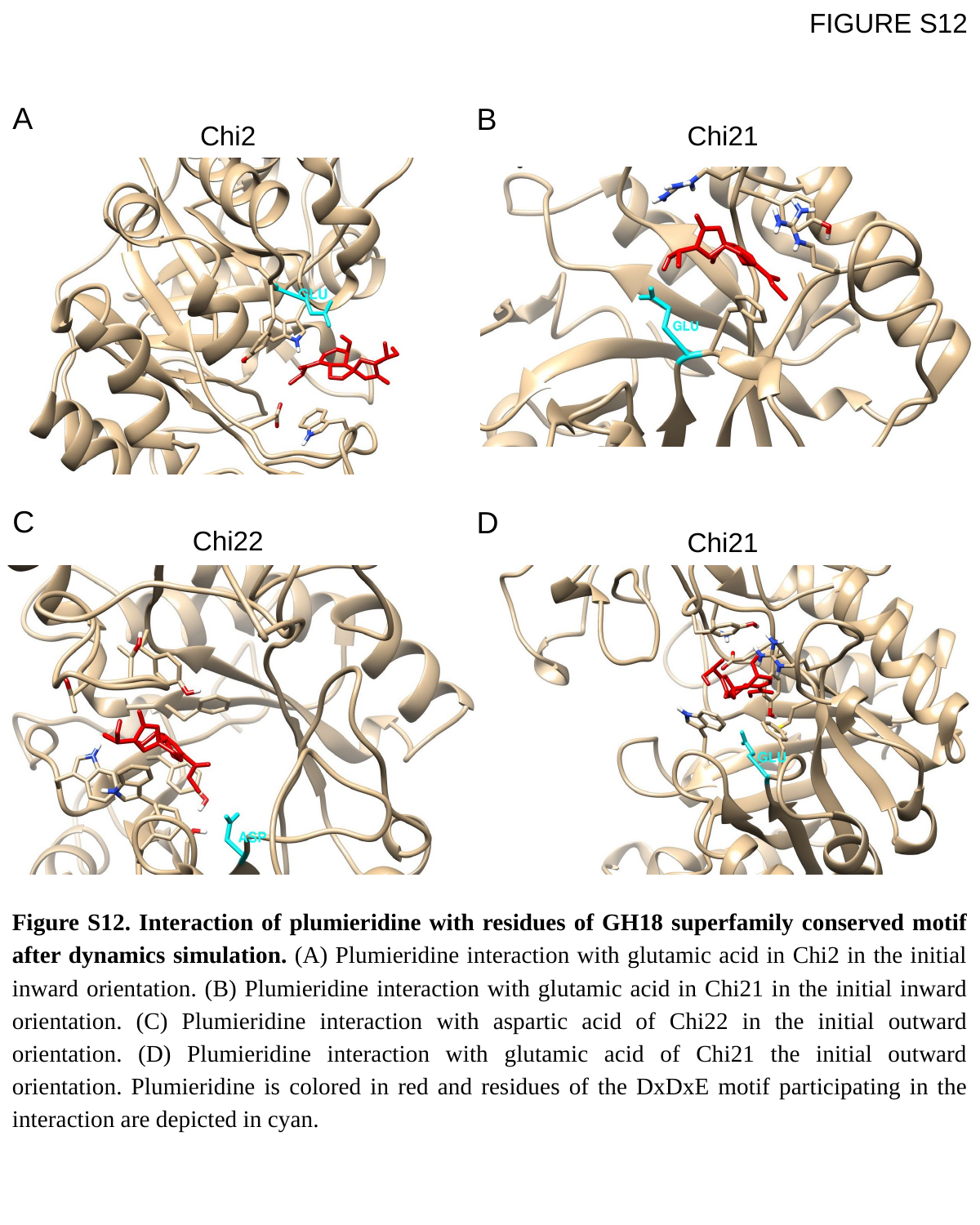

FIGURE S12
A
B
Chi2
Chi21
C
D
Chi22
Chi21
Figure S12. Interaction of plumieridine with residues of GH18 superfamily conserved motif after dynamics simulation. (A) Plumieridine interaction with glutamic acid in Chi2 in the initial inward orientation. (B) Plumieridine interaction with glutamic acid in Chi21 in the initial inward orientation. (C) Plumieridine interaction with aspartic acid of Chi22 in the initial outward orientation. (D) Plumieridine interaction with glutamic acid of Chi21 the initial outward orientation. Plumieridine is colored in red and residues of the DxDxE motif participating in the interaction are depicted in cyan.

## Slide 13
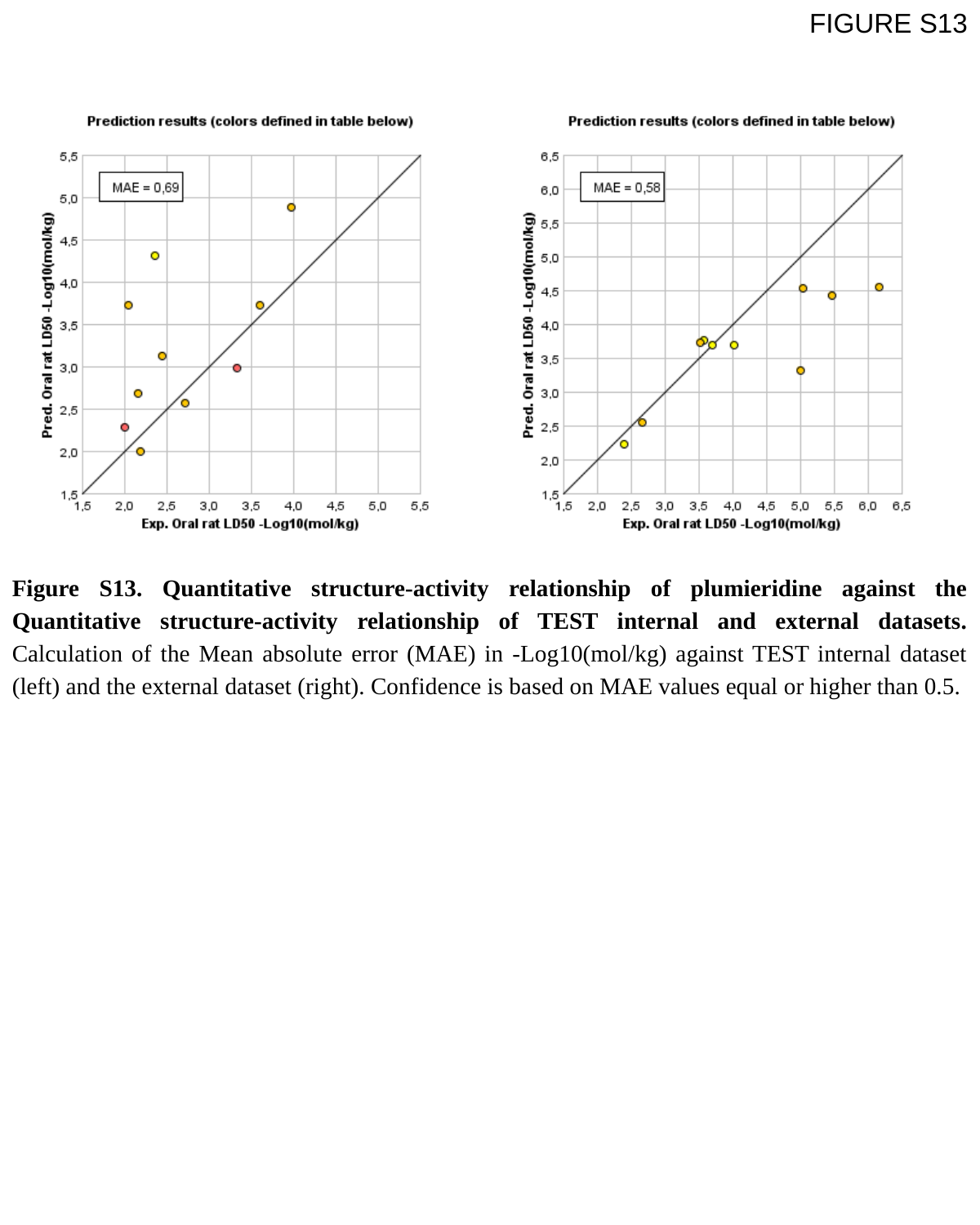

FIGURE S13
Figure S13. Quantitative structure-activity relationship of plumieridine against the Quantitative structure-activity relationship of TEST internal and external datasets. Calculation of the Mean absolute error (MAE) in -Log10(mol/kg) against TEST internal dataset (left) and the external dataset (right). Confidence is based on MAE values equal or higher than 0.5.
